# Effect of organic fertilizer substitution on soil fertility and yield in a continuous maize cropping system: evidence from an eight-year long-term field experiment

**DOI:** 10.3389/fpls.2026.1824928

**Published:** 2026-04-21

**Authors:** Xiaogang Liu, Chengbao Wang, Youshuai Bai, Lin Huo, Meijuan Wen, Mingcong Wang

**Affiliations:** 1 Institute of Soil, Fertilizer and Water-saving Agriculture, Gansu Academy of Agricultural Sciences, Lanzhou, Gansu, China; 2 National Agricultural Science Baiyin Observation and Experimental Station, Baiyin, Gansu, China; 3 College of Resources and Environmental Sciences, Gansu Agricultural University, Lanzhou, Gansu, China

**Keywords:** arid irrigated area, grain yield, maize physiological indicators, sierozem, soil nutrients

## Abstract

**Introduction:**

Long-term fertilization regimes strongly influence soil fertility and carbon–nutrient cycling in cropland ecosystems. However, in calcareous soils of arid irrigated regions in north-western China, evidence remains limited on how different fertilization practices affect canopy function and soil CO_2_ exchange during maize grain filling.

**Methods:**

Based on an eight-year field experiment at the National Agricultural Science Baiyin Observation and Experimental Station, this study evaluated the effects of different fertilization regimes on soil fertility, canopy function, soil net CO_2_ exchange rate (NCER), and maize yield in a continuous maize cropping system. Four treatments with equivalent nutrient inputs were established: no fertilization (CK), conventional chemical fertilization (NP), one-third substitution of chemical fertilizer with commercial organic fertilizer (1/3M), and sole application of commercial organic fertilizer (M). Leaf photosynthetic traits, canopy structure, soil water content, and soil NCER were measured at the early, middle, and late grain-filling stages, while soil physicochemical properties, yield components, and plant nutrient contents were determined after harvest.

**Results:**

Fertilization significantly altered the nutrient pool structure of the Sierozem. Compared with CK, NP markedly increased soil available phosphorus and total phosphorus, whereas M significantly increased soil organic matter, total nitrogen, and available potassium, but also raised soil electrical conductivity. By contrast, 1/3M achieved a more coordinated improvement in soil nutrient status while maintaining stable soil pH. Although canopy function gradually declined with grain-filling progression, NP and 1/3M maintained significantly higher photosynthetic capacity than CK during the middle and late stages. Soil NCER showed clear stage-dependent variation, peaking at the middle grain-filling stage, and was significantly enhanced by fertilization, with 1/3M increasing NCER by 107.3% at the middle stage and by 145.6% at the late stage relative to CK. Maize yield was highest under 1/3M, increasing by 143.0% relative to CK.

**Discussion:**

Overall, canopy function during grain filling was closely coordinated with soil NCER and yield formation, and one-third substitution of chemical fertilizer with organic fertilizer was the most effective strategy for improving soil fertility, sustaining canopy function, stabilizing carbon processes, and increasing yield.

## 1 Introduction

Improving cropland quality and achieving high and stable crop yields are central strategic tasks for safeguarding national food security and advancing sustainable agricultural development in China. They also represent key challenges for dryland agricultural regions worldwide in responding to climate change and soil degradation (Food Agriculture Organization, 2017, Wang et al., 2025b). The arid and semi-arid regions of north-western China constitute an important ecological barrier and strategic reserve for grain production in northern China. Irrigated agriculture is the core of grain production in this region, and the stability of cropland quality together with the sustainable improvement of crop productivity is directly linked to regional ecological security and national grain supply security (Xin, 2022). Maize is the dominant cereal crop in the Yellow River irrigated area of Gansu Province, accounting for more than 60% of the total grain-sown area in the region. It is the principal crop for increasing farmers’ income and optimizing the local planting structure, and its high and stable productivity depends heavily on water lifted from the Yellow River for irrigation and on exogenous nutrient inputs (Li et al., 2024; Hao and Zhou, 2025). For a long time, maize production in this region has generally relied on a fertilization pattern characterized by heavy use of chemical fertilizers and limited application of organic fertilizers. Although this practice has increased yields in the short term, many years of continuous and exclusive chemical fertilizer use have resulted in a series of cropland quality degradation problems, including declines in soil organic matter, nutrient imbalance, structural deterioration, and reduced microbial activity (Li et al., 2021). At the same time, it has intensified imbalances in carbon cycling within farmland ecosystems, thereby further constraining the sustainable improvement of maize productivity (Geisseler and Scow, 2014, Khan et al., 2022a, Tang et al., 2025).

Fertilization is a core agronomic management practice for regulating soil fertility, crop productivity, and agro-ecological effects, and it is also critical for coordinating food security with ecological security (Shi et al., 2024). Conventional chemical fertilizers can rapidly supply readily available nutrients required for crop growth and are therefore essential for ensuring high and stable maize yields. However, long-term excessive application can promote phosphorus fixation through the formation of calcium phosphate complexes in calcareous soils, thereby reducing nutrient use efficiency (Zhang et al., 2025). It may also disrupt the structure of water-stable aggregates and weaken the soil’s capacity for water and nutrient retention (Lin et al., 2025). In arid irrigated areas, it can further aggravate the risk of salt accumulation in the surface soil layer (Mohanavelu et al., 2021). Substituting chemical fertilizer with organic fertilizer, as a key technology promoted for green agricultural development in China, has been widely validated across multiple agricultural regions. Inputs of exogenous organic fertilizer can enhance soil organic carbon content and activity through continuous carbon replenishment, improve soil pore structure and aggregate stability, strengthen soil water- and nutrient-holding capacity, and increase microbial functional diversity, thereby promoting long-term soil fertility improvement (Imran et al., 2021; Salman et al., 2023; Guo et al., 2022; Lin et al., 2025). Meanwhile, the combined use of organic and chemical fertilizers can achieve synergy between rapid nutrient supply and sustained nutrient release, more precisely matching the nutrient demand of maize throughout the entire growth period, effectively delaying the senescence of functional leaves during late growth stages, and improving crop yield and nutrient use efficiency (Fan et al., 2021; Deng et al., 2023). Numerous studies have shown that an organic fertilizer substitution ratio of 20%-50% can simultaneously achieve higher crop yields, soil fertility improvement, and greenhouse gas mitigation, making it an optimal fertilization pattern that balances production and ecological benefits (Fan et al., 2021; Shi et al., 2024, Wang et al., 2025a).

Although the yield-increasing and soil-improving effects of substituting chemical fertilizer with organic fertilizer have been widely demonstrated, most existing studies have focused on static responses of soil physicochemical properties and final yield at harvest, with insufficient attention given to the dynamic physiological processes during the grain-filling period, a critical stage for maize yield formation (Wang et al., 2024). A large body of research has shown that 60%-80% of maize grain yield is derived from photosynthates produced by functional leaves around the ear during grain filling, and the duration and activity of canopy photosynthetic function maintained during the middle and late grain-filling stages directly determine final grain weight and yield (Deng et al., 2023). However, few studies have systematically examined the regulatory effects of organic fertilizer substitution on canopy structure and the decline in photosynthetic physiology across different stages of maize grain filling, particularly under the coordinated influence of soil water and nutrient availability in arid irrigated regions (Guo et al., 2021). In addition, existing studies on the effects of fertilization on soil CO_2_ exchange have mainly concentrated on mean fluxes over the whole crop growth period or on emission characteristics during non-growing seasons, with very limited effort devoted to integrating soil carbon processes with aboveground crop photosynthetic physiology (Guo et al., 2021). The maize grain-filling period is the peak stage for photosynthetic carbon assimilation and belowground carbon allocation. Approximately 30%-60% of the carbon fixed by plants is transferred to rhizosphere soil through root exudation and residue inputs, thereby acting as a key driver of soil CO_2_ exchange (Hirte et al., 2018). Nevertheless, the coordinated regulatory mechanisms linking these processes and their responses to fertilization management remain unclear. This knowledge gap is particularly evident in carbonate-rich Sierozems, where carbonate dissolution-precipitation processes can substantially interfere with soil CO_2_ fluxes, yet related studies remain scarce (Cai et al., 2018; Sun et al., 2023). In contrast, the alkaline conditions of Sierozems can strongly influence phosphorus availability, micronutrient supply, and plant nutrient uptake, making fertilization management in these soils fundamentally different from that in other major agricultural soil types (Brownrigg et al., 2022; Bontpart et al., 2024). Moreover, current studies on organic fertilizer substitution have mainly focused on representative agricultural regions in China, such as the black soils of north-eastern China, the fluvo-aquic soils of the North China Plain, and the red soils of southern China (Fan et al., 2021; Shi et al., 2024). By contrast, long-term field studies remain limited for the continuous maize cropping system under border irrigation on Sierozems in the Yellow River irrigated area of north-western China (Sun et al., 2023; Li et al., 2024). As a typical calcareous soil in arid regions, Sierozem is characterized by carbonate accumulation, high pH, strong acid-base buffering capacity, and nutrient transformation processes that differ markedly from those in acidic soils. Under border irrigation, this soil is also subject to the combined risks of salt accumulation and nutrient leaching. Therefore, conclusions drawn from other regions cannot be directly applied to the production realities of this area, and long-term field evidence is still insufficient.

Against this background, the present study was conducted using a long-term fertilization experiment established in 2018, with a border-irrigated continuous maize cropping system on Sierozem in the Yellow River irrigated area of Gansu Province as the study object. Stage-specific systematic observations were carried out during maize grain filling to address the following scientific questions: (1) what are the dynamic differences in soil NCER and its temperature sensitivity across different grain-filling stages under different fertilization regimes; (2) how do canopy function maintenance and soil water content drive stage-dependent variation in NCER; and (3) how does long-term fertilization regulate soil nutrient pool structure and crop nutrient allocation, ultimately affecting yield formation? We hypothesized that one-third substitution of chemical fertilizer with organic fertilizer would improve soil fertility and the soil water environment while ensuring an adequate supply of readily available nutrients, thereby more effectively sustaining canopy activity during the middle and late grain-filling stages, enhancing the coordination of carbon processes within the plant-soil system, promoting grain filling, and achieving both stable yield increases and improved soil quality. The results of this study are expected to provide long-term field evidence and scientific support for fertilization optimization and cropland quality improvement in Sierozem-based farmland systems in the Yellow River irrigated area of north-western China.

## 2 Materials and methods

### 2.1 Overview of the experimental site

The experiment was conducted in Jingtan Village, Beitan Town, Jingyuan County, Baiyin City, Gansu Province, China (37°05′N, 104°40′E), within a Yellow River lift-irrigation district. Irrigation water in this district is supplied by lifting water from the Yellow River by approximately 480 m. The experimental site is located at an altitude of 1,645 m above sea level, on the northern side of the transition zone from dryland agriculture to wasteland-pasture, and belongs to the arid loess hilly and gully region. The climate is arid, with a mean annual precipitation of 259 mm and a mean annual evaporation of 2,369 mm. The mean annual air temperature is 6.6 °C, and the accumulated temperatures above 0 °C and 10 °C are 3,208 °C and 2,622 °C, respectively. The frost-free period lasts 160–170 days. The region receives 2,919 h of sunshine annually, with a total annual solar radiation of 616.2 kJ cm⁻². Farmland irrigation water is derived from Yellow River lift-irrigation, and border irrigation is used.

The soil at the experimental site is classified as a Sierozem, with a medium loam texture and alluvial loess as the parent material. Before the experiment was established, the field had long been managed under conventional tillage and continuously planted with maize. Before sowing, the basic physicochemical properties of the 0–20 cm plough layer were as follows: soil organic matter, 12.58 g kg⁻¹; total nitrogen, 1.22 g kg⁻¹; total phosphorus, 1.09 g kg⁻¹; total potassium, 1.35 g kg⁻¹; alkali-hydrolysable nitrogen, 45.4 mg kg⁻¹; available phosphorus, 11.5 mg kg⁻¹; available potassium, 193 mg kg⁻¹; pH 8.25; bulk density, 1.43 g cm⁻³; and calcium ion (Ca²^+^) content, 0.18–0.24 g kg⁻¹. The experiment was initiated in 2018 under a continuous maize cropping system.

### 2.2 Experimental design

The experiment was initiated in the spring of 2018 under a continuous maize cropping system. Four fertilization treatments were established: (1) no fertilization (CK); (2) conventional chemical fertilization (NP); (3) optimized fertilization with one-third substitution by organic fertilizer (1/3M); and (4) sole application of organic fertilizer (M). A randomized block design was adopted, with three replicates for each treatment. The plot area was 128.7 m².

The recommended doses of nitrogen (N) and phosphorus (P) were supplied using urea and diammonium phosphate, respectively. Specifically, nitrogen was provided by both urea and diammonium phosphate, whereas phosphorus was supplied by diammonium phosphate. No potassium fertilizer was applied in this experiment. The total inputs of N and P were kept identical across all fertilized treatments. In the NP treatment, fertilizer was applied at rates of 375 kg N hm⁻² and 65.5 kg P hm⁻². Nitrogen fertilizer was applied in split doses, with 40% as a basal application and 60% top-dressed at the jointing stage in combination with irrigation, whereas phosphorus fertilizer was applied entirely as a basal dressing. In the 1/3M treatment, commercial organic fertilizer was used to substitute part of the chemical fertilizer input at the target nutrient input level, such that one-third of the total N and P inputs was supplied by organic fertilizer and the remaining two-thirds by chemical fertilizer. The organic fertilizer and phosphorus fertilizer were applied entirely as basal fertilizers, while nitrogen fertilizer was still applied according to the same basal and top-dressing ratio. In the M treatment, only commercial organic fertilizer was applied, and the application rate was calculated according to its nutrient content to meet the predetermined N and P input targets. According to the product label, the total nutrient content of the commercial organic fertilizer (N + P_2_O_5_ + K_2_O) was ≥4%, organic matter ≥30%, and moisture ≤30%. No fertilizer was applied in the CK treatment. Apart from the fertilization regime, all other field management practices were consistent across treatments and followed locally recommended agronomic practices.

The test crop was maize (*Zea mays* L., cv. Xianyu 1483), planted at a density of 6.75 × 10^4^ plants hm⁻². Maize was generally sown in mid-April and harvested in early October each year. Border irrigation was adopted, and irrigation was applied four times during the entire growth period, with a total irrigation quota of approximately 5,400 m³ hm⁻². The proportions of irrigation water applied during the emergence-jointing, jointing-tasselling, tasselling-milking, and milking-maturity stages were 16%, 28%, 31%, and 25%, respectively. Intertillage, weeding, and pest and disease control after treatment implementation were all managed in accordance with local field production practices.

### 2.3 Measurements and calculations

#### 2.3.1 Soil sampling and sample processing

Soil samples and maize physiological growth indicators were measured in 2025. Soil samples were collected from the 0–20 cm plough layer using a five-point sampling method. The soil collected from the five sampling points within each plot was thoroughly mixed to form a composite sample. The samples were transported to the laboratory, naturally air-dried, cleared of plant residues and other impurities, ground, and sieved for the determination of soil nutrient properties (Xu et al., 2026). Soil organic matter (OM) was determined by the potassium dichromate oxidation-heating method according to NY/T 1121.6-2006. Soil available phosphorus (AP) was determined according to HJ 704-2014, soil available potassium (AK) according to LY/T 1234-2015, soil hydrolyzable nitrogen (HN) according to LY/T 1228-2015, soil total potassium (TK) according to NY/T 87-1988, soil total nitrogen (TN) according to NY/T 53-1987, soil total phosphorus (TP) according to GB/T 9837-1988, soil pH according to NY/T 1121.2-2006, total soil water-soluble salts according to NY/T 1121.16-2006, and soil cation exchange capacity (CEC) according to NY/T 1121.5-2006. Measurements during the maize grain-filling period were conducted at three stages: early grain filling (28 July), middle grain filling (16 August), and late grain filling (2 September).

Maize plant height was measured using a plant height ruler (cm). Five plants were randomly selected from each plot for measurement, and the mean value of three replicates was used for statistical analysis. Leaf area index (LAI) and photosynthetically active radiation (PAR) were measured at the base of the maize canopy using a SunScan canopy analysis system. Eight measurements were taken in each plot, and the mean value was used as the observation for that plot at each stage. Leaf photosynthetic parameters were measured at the three grain-filling stages between 08:00 and 12:00 using a portable photosynthesis system (GFS-3000), including net photosynthetic rate (A) and transpiration rate (E). In each plot, functional leaves at the ear position were selected from plants with uniform growth, and eight plants were measured per plot. The mean values of A and E for each plot were used for statistical analysis. Relative chlorophyll content (SPAD) was measured synchronously at the same stages using a Konica Minolta SPAD-502 Plus chlorophyll meter on the same functional leaves used for gas-exchange measurements. Eight plants were measured in each plot, and the mean value was taken as the SPAD observation for that plot.

Soil NCER was determined using an SRS 2000T portable soil respiration system at the same three stages of the maize grain-filling period. Twelve hours before measurement, a soil collar (10 cm in diameter and 10 cm in height) was installed in the selected soil area using the rigid insertion tool provided with the instrument. The metal plate was placed on top of the collar, and the collar was gently inserted into the soil by foot pressure or with a rubber mallet until the soil surface inside the collar was just below the top edge of the chamber. All living plants within the collar base were removed carefully to avoid root damage. During measurement, NCER and soil temperature were continuously recorded for 10 min at each observation point in each plot. Soil water content was determined by the oven-drying method. Soil samples were collected simultaneously from the 0–20 cm soil layer at the same positions where soil NCER was measured, returned to the laboratory, oven-dried to constant weight, and then used to calculate soil water content (Czubaszek, 2019).

After maize maturity, each plot was harvested separately on 9 October. In each plot, 10 plants were sampled for yield component analysis, and one entire row was harvested to determine grain yield and aboveground biomass. For nutrient analysis, plant samples were collected from the plants used for yield assessment in each plot. After separation into grain and straw, the samples were processed and transported to the laboratory for the determination of total N, P, and K concentrations (Guo et al., 2024). Plant total nitrogen was determined by the automatic Kjeldahl method according to NY/T 2419-2013, plant total potassium by flame photometry according to NY/T 2420-2013, and plant total phosphorus by the molybdenum-antimony anti-colorimetric method according to NY/T 2421-2013. Each treatment included three replicate plots. Indicators including LAI, PAR, A, E, SPAD, NCER, and soil water content were measured repeatedly at the plot scale, and the mean value for each plot was calculated. Data from the three replicate plots under each treatment were then pooled to obtain treatment means for subsequent statistical comparisons, correlation analysis, and multivariate analysis.

#### 2.3.2 Calculation methods

1. Soil CO_2_ exchange rate (NCER).

NCER was expressed as the concentration change output by the instrument and fitted to soil temperature using the following exponential model (Equation 1), according to (Zhou et al., 2013):

(1) lnR=lna+bT

where *R* is the NCER, *T* is soil temperature (°C), *a* is the soil respiration rate at 0 °C, and *b* is the temperature response coefficient.

The temperature sensitivity coefficient (*Q*_10_) was calculated as follows (Equation 2):

(2) $$Q_{10}=e^{10b}$$

2. Yield calculation.

The grain moisture content was corrected to the standard moisture content of 14% using Equation 3 (Cao et al., 2024):

(3) $$W_{14}=W_{f}\times \frac{1-M_{f}}{1-14\%}$$

where W_14_ is the standard grain weight at 14% moisture content in the harvested area (kg), W_f_ is the measured fresh grain weight in the harvested area (kg), and M_f_ is the measured grain moisture content in decimal form.

The standard grain yield per plot was calculated as follows (Equation 4):

(4) $$Y_{plot}=W_{14}\times 9$$

where Yplot is the grain yield of the entire plot at 14% standard moisture content (kg), and 9 is the fixed area conversion coefficient, because the harvested area accounted for one-ninth of the total plot area.

The harvest index (HI) was calculated from the grain yield and biological yield of 10 maize plants (Equation 5):

(5) $$HI=\frac{Y_{grain}}{Y_{biomass}}$$

where Y_grain_ is the grain yield of 10 maize plants (kg), and Y_biomass_ is the biological yield of 10 maize plants (kg).

The aboveground biomass yield of maize was calculated as follows (Equation 6):

(6) $$Y_{biomass,plot}=\frac{Y_{\text{plot}}\times (1-14\%)}{HI}$$

where Y_biomass,plot_ is the total aboveground oven-dry biomass yield of maize in the whole plot (kg), and Y_plot_ × (1 − 14%) is the oven-dry grain weight per plot.

The final yield per hectare was calculated as follows (Equation 7):

(7) $$Y_{final}=\frac{Y_{plot}}{s_{plot}}\times 10$$

where Y_final_ is the final grain yield per hectare (t hm⁻²), Y_plot_ is the standard grain yield per plot (kg), S_plot_ is the total plot area (128.7 m²), and 10 is the fixed unit conversion coefficient based on 1 hm² = 10,000 m² and 1 t = 1,000 kg.

For yield component analysis, 10 representative plants were sampled from each plot. The 100-grain weight was calculated based on grain counting and weighing, corrected to 14% moisture content, as follows (Equation 8):

(8) $$W_{100}=\frac{W_{k}\times (1-M_{k})}{\left(1-14\%\right)}$$

where W_100_ is the 100-grain weight (g), W_k_ is the actual weight of 100 grains (g), and M_k_ is the moisture content of the counted grain sample (%).

### 2.4 Statistical analyses

Data processing and statistical analyses were performed using Microsoft Excel 2021, R 4.3.2, Origin 2025, and IBM SPSS 22.0. Excel was used for raw data organization, summarization, and preliminary calculations; R 4.3.2 was used for correlation analysis, multivariate statistical analysis, and visualization; Origin 2025 was used for figure preparation and layout; and IBM SPSS 22.0 was used for analysis of variance. Plot was used as the statistical unit, and data from the three replicate plots under each treatment were used for statistical testing. Results are presented as mean values. Differences among treatments were assessed by one-way analysis of variance, and *post hoc* multiple comparisons were performed using the least significant difference (LSD) test at the *p* < 0.05 level.

## 3 Results

### 3.1 Effects of fertilization treatments on the physicochemical properties of Sierozem

**Figure 1:**
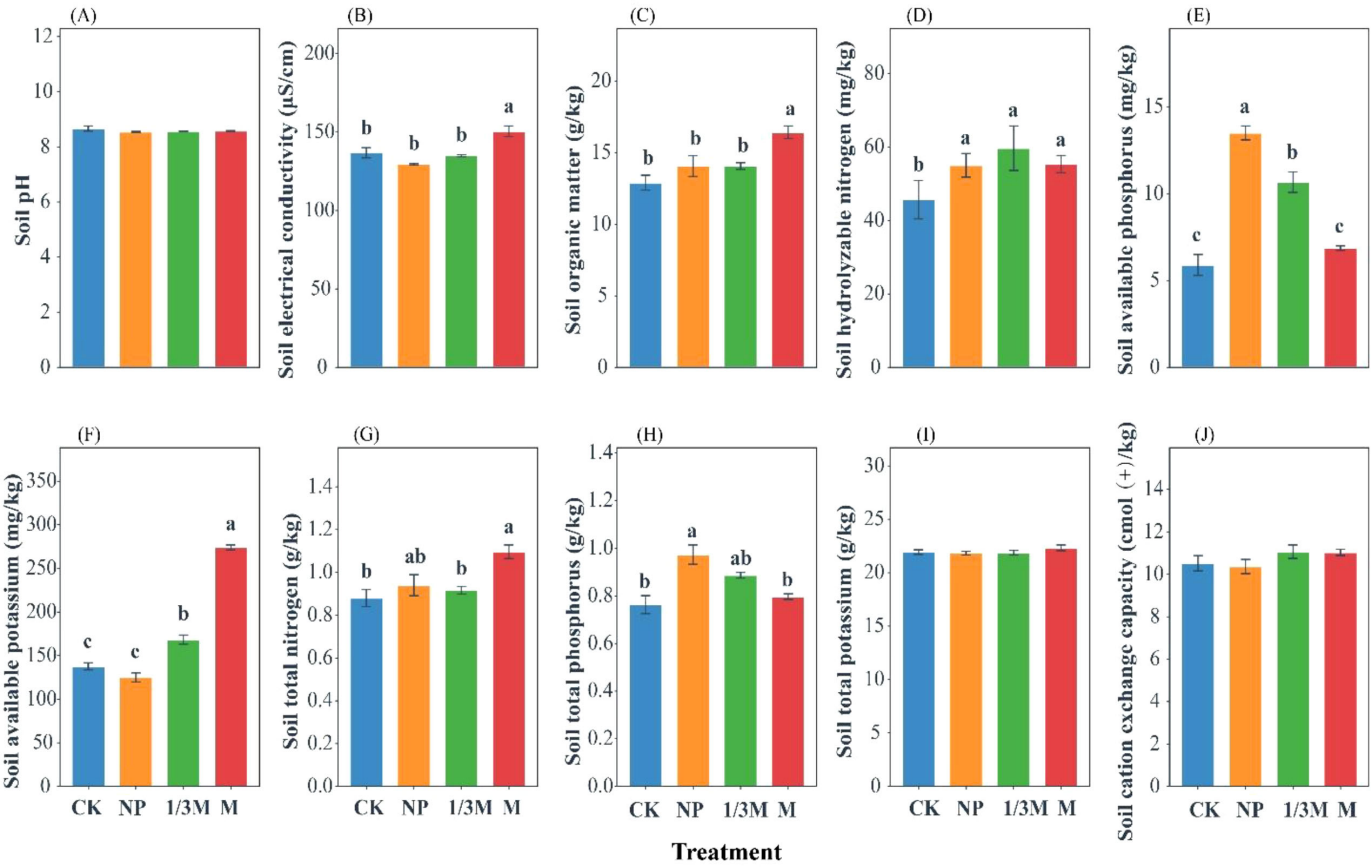
Effects of different fertilization treatments on soil physicochemical properties in grey calcareous soil: CK, no fertilization; NP, conventional chemical fertilization; 1/3M, optimized fertilization with one-third substitution by organic fertilizer; M, sole application of organic fertilizer. **(A)** soil pH; **(B)** soil electrical conductivity; **(C)** soil organic matter; **(D)** soil hydrolyzable nitrogen; **(E)** soil available phosphorus; **(F)** soil available potassium; **(G)** soil total nitrogen; **(H)** soil total phosphorus; **(I)** soil total potassium; and **(J)** soil cation exchange capacity. Bars represent the mean ± standard error. Different lowercase letters indicate significant differences among treatments at *P* < 0.05.

As shown in **Figure 1**, different fertilization treatments significantly affected soil physicochemical properties and nutrient status. Soil pH ranged from 8.54 to 8.67 across treatments and remained relatively stable. Compared with CK, the M treatment significantly increased electrical conductivity (EC) and organic matter (OM) by 10.04% and 27.36%, respectively. The NP treatment resulted in the highest levels of available phosphorus (AP) and total phosphorus (TP), with AP and TP being significantly increased by 128.8% and 27.52%, respectively, compared with CK. In the 1/3M treatment, AP was significantly increased by 80.85% relative to CK. The M treatment significantly increased available potassium (AK) by 99.03% compared with CK and showed a significantly higher value than the other treatments, whereas the 1/3M treatment increased AK by 22.27% relative to CK. The M treatment significantly increased total nitrogen (TN) by 24.60% compared with CK. In addition, hydrolyzable nitrogen (HN) was significantly higher in the NP, 1/3M, and M treatments than in CK, with increases of 20.44%, 30.66%, and 21.17%, respectively, although no significant differences were observed among these three treatments. By contrast, total potassium (TK) and cation exchange capacity (CEC) did not differ significantly among treatments.

**Figure 2:**
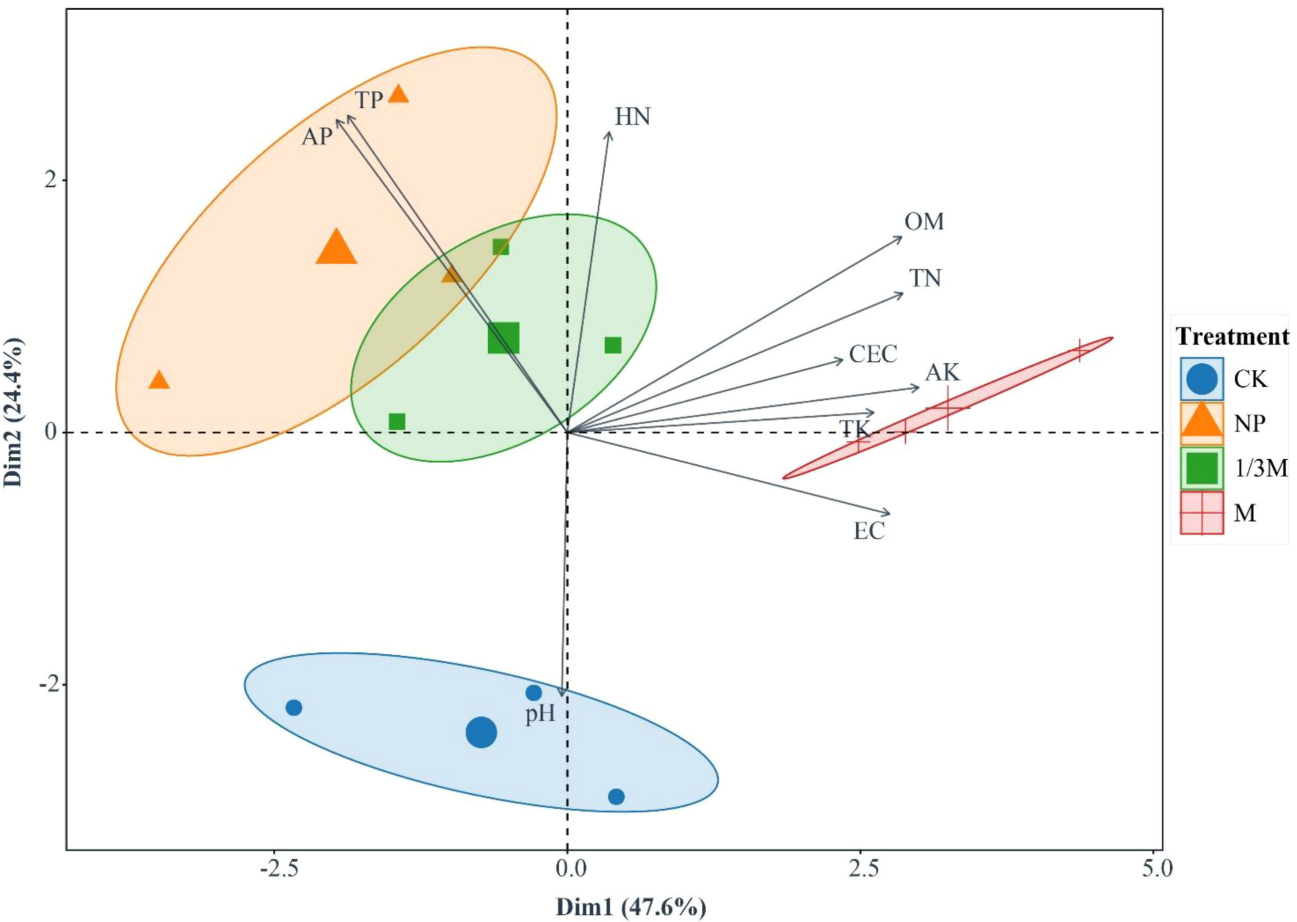
PCA biplot of soil nutrients and key physicochemical properties under different fertilization treatments: CK, no fertilization; NP, conventional chemical fertilization; 1/3M, optimized fertilization with one-third substitution by organic fertilizer; M, sole application of organic fertilizer. OM, soil organic matter; HN, soil hydrolyzable nitrogen; TN, soil total nitrogen; TP, soil total phosphorus; AP, soil available phosphorus; AK, soil available potassium; TK, soil total potassium; CEC, soil cation exchange capacity; EC, soil electrical conductivity; pH, soil pH. Ellipses indicate the distribution of replicates within each treatment group.

Principal component analysis (PCA) showed a clear separation among fertilization treatments in the two-dimensional space (**Figure 2**). The first two axes (Dim1 = 47.6% and Dim2 = 24.4%) together explained 72.0% of the total variance, indicating distinct differences among fertilization treatments. The variable loadings showed that Dim1 was mainly positively associated with OM, TN, CEC, AK, TK, and EC, whereas Dim2 was more closely aligned with AN, AP, and TP. The distribution of sample points showed that the M treatment was located mainly on the positive side of Dim1 and was oriented in the same direction as the vectors for OM, TN, AK, and EC, indicating that its soil characteristics were closely associated with increased soil organic matter, total nitrogen, available potassium, and electrical conductivity. The NP treatment was positioned towards the negative side of Dim1 and the positive side of Dim2, and was more consistent with the directions of the AP and TP vectors, reflecting that its main characteristic was an increase in phosphorus levels. The 1/3M treatment was located between M and NP, showing a pattern of improved nutrient status with relatively moderate magnitude. By contrast, the CK treatment was concentrated on the negative side of Dim2 and was closer to the direction of the pH vector, indicating that in the absence of exogenous nutrient inputs, soil properties were more strongly controlled by the inherent physicochemical characteristics of the soil.

### 3.2 Effects of different fertilization treatments on canopy structure, physiological traits, and soil water content during the maize grain-filling period

#### 3.2.1 Plant height

**Figure 3:**
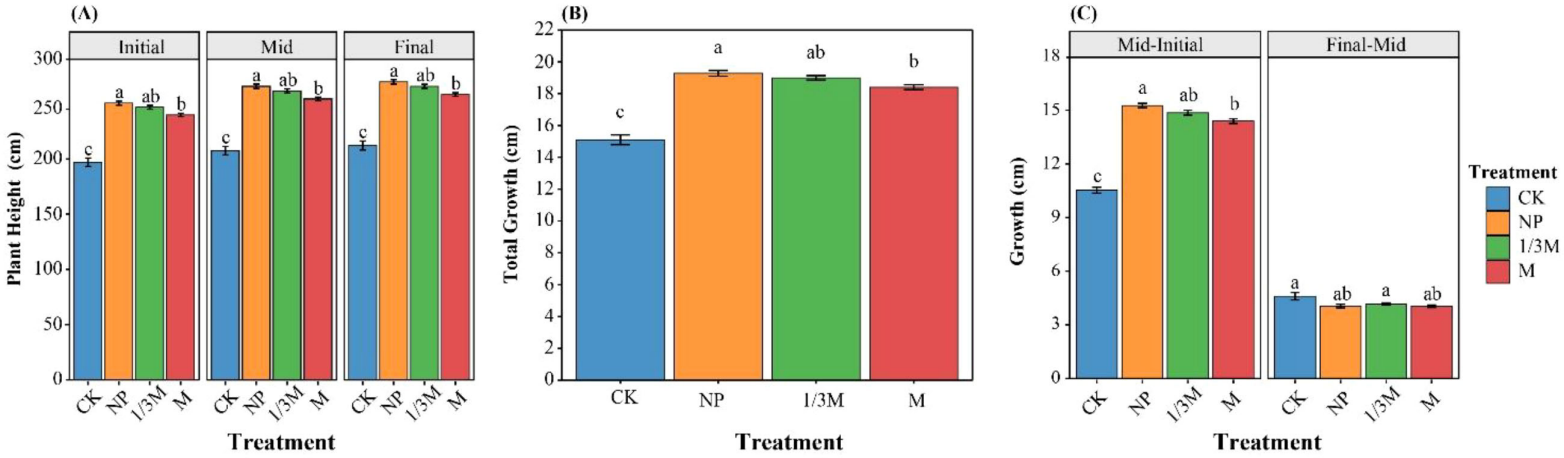
Changes in maize plant height at different growth stages under different fertilization treatments: **(A)** Plant height at the initial, middle, and final grain-filling stages; **(B)** total growth from the initial to the final stage; and **(C)** growth increments from the initial to the middle stage and from the middle to the final stage. CK, no fertilization; NP, conventional chemical fertilization; 1/3M, optimized fertilization with one-third substitution by organic fertilizer; M, sole application of organic fertilizer. Bars represent the mean ± standard error. Different lowercase letters indicate significant differences among treatments within the same period at *p* < 0.05.

Across the different growth stages, the ranking of maize plant height among fertilization treatments remained generally consistent, following the order NP ≥ 1/3M > M > CK (**Figure 3**). At the early grain-filling stage, plant height under all fertilization treatments was greater than that under CK, with NP and 1/3M showing the highest values and M ranking second. Compared with CK, plant height increased significantly by 27.14%, 25.25%, and 21.76% under the NP, 1/3M, and M treatments, respectively. At the middle grain-filling stage, plant height under NP, 1/3M, and M was significantly higher than that under CK by 28.04%, 26.05%, and 22.52%, respectively. At the late grain-filling stage, the corresponding increases were 27.17%, 25.28%, and 21.77%, respectively. The increment in plant height (**Figure 3B**) indicated that growth was mainly concentrated between the early and middle grain-filling stages. Plant height in CK increased by 10.53 cm, whereas the increases under NP, 1/3M, and M were 15.27, 14.87, and 14.40 cm, respectively. From the middle to late grain-filling stage, the increase in plant height was generally small across all treatments, ranging from 4.0 to 4.6 cm, and the differences among treatments became much less pronounced.

#### 3.2.2 Leaf SPAD value

**Figure 4:**
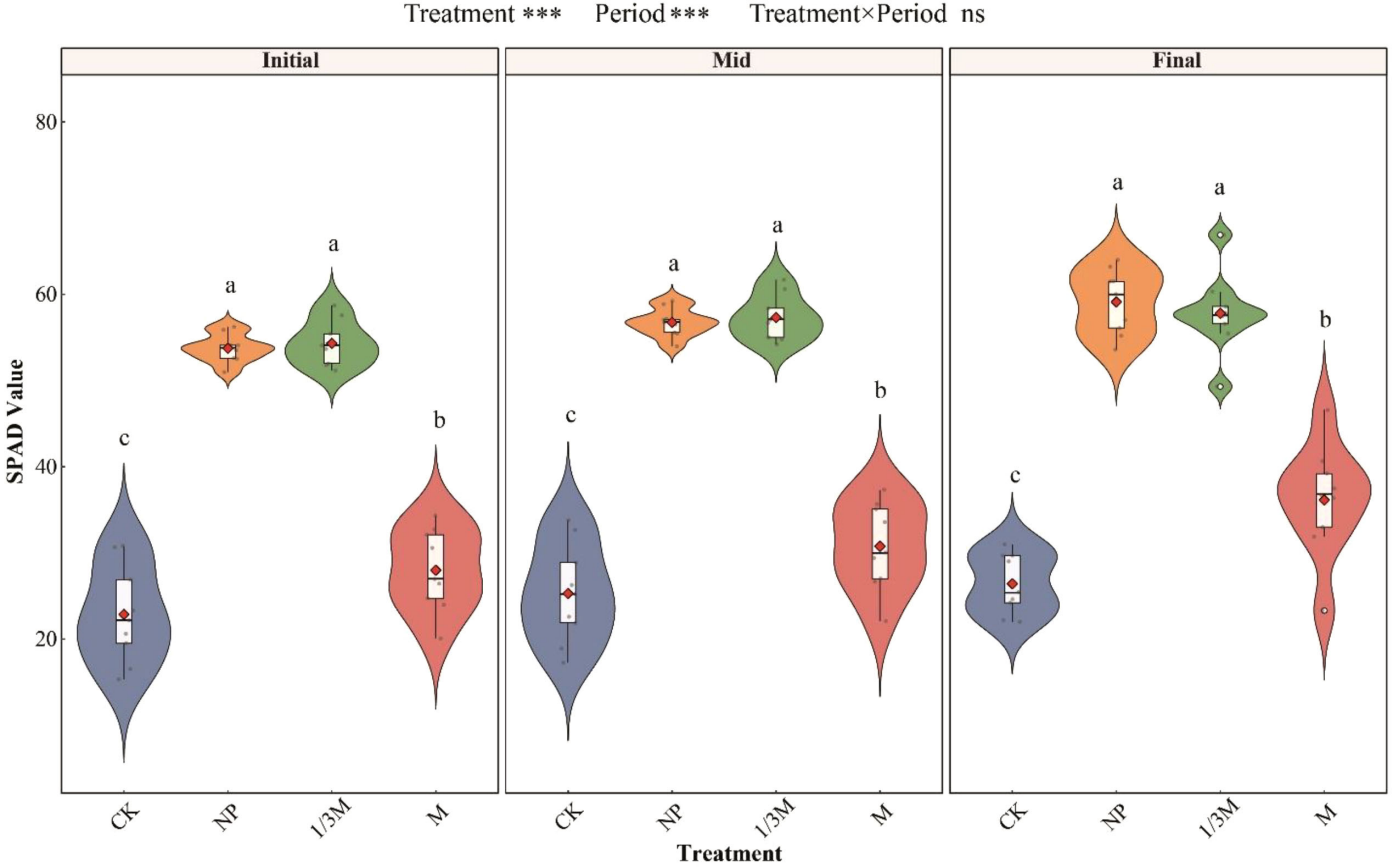
Changes in SPAD values of maize leaves at different growth stages under different fertilization treatments. *p < 0.05; **p < 0.01; ***p < 0.001; ns denotes no significant: CK, no fertilization; NP, conventional chemical fertilization; 1/3M, optimized fertilization with one-third substitution by organic fertilizer; M, sole application of organic fertilizer. Initial, early grain filling; Mid, middle grain filling; Final, late grain filling. Different lowercase letters indicate significant differences among treatments within the same period at *p* < 0.05. Asterisks above the panels indicate the significance of treatment, period, and treatment × period interaction; ns, not significant; ***p < 0.001.

Across the three grain-filling stages (**Figure 4**), the relative chlorophyll content (SPAD) showed a relatively consistent pattern among treatments. The NP and 1/3M treatments consistently produced the highest SPAD values at the early, middle, and late grain-filling stages, with significant increases of 118.8%–137.5% compared with CK. These two treatments also showed the greatest and most stable enhancement in SPAD values. The M treatment significantly increased SPAD by 21.66%–36.80% relative to CK, although the magnitude of increase was lower than that under NP and 1/3M. Overall, NP and 1/3M showed the most stable and pronounced effects on maintaining leaf chlorophyll during the maize grain-filling period, followed by M, whereas CK consistently showed the lowest SPAD values.

#### 3.2.3 Leaf area index and photosynthetically active radiation

**Figure 5:**
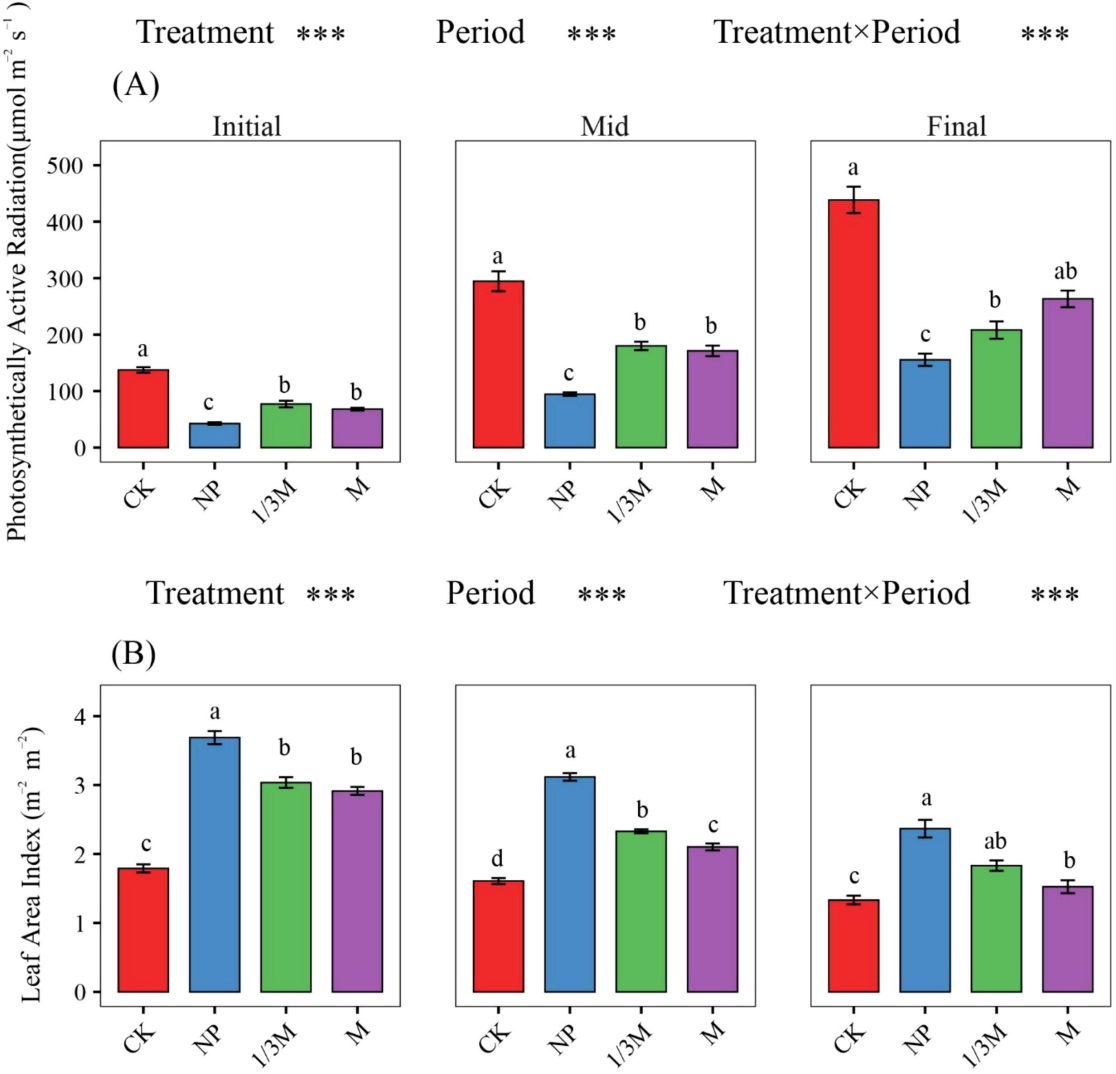
Changes in leaf area index (LAI) and photosynthetically active radiation (PAR) at different growth stages under different fertilization treatments: **(A)** Photosynthetically active radiation at the initial, middle, and final grain-filling stages; **(B)** leaf area index at the initial, middle, and final grain-filling stages. CK, no fertilization; NP, conventional chemical fertilization; 1/3M, optimized fertilization with one-third substitution by organic fertilizer; M, sole application of organic fertilizer. Bars represent the mean ± standard error. Different lowercase letters indicate significant differences among treatments within the same period at *p* < 0.05. Asterisks above the panels indicate the significance of treatment, period, and treatment × period interaction; ***p < 0.001.

At the early grain-filling stage (**Figure 5**), compared with CK, LAI increased by 105.8%, 69.42%, and 62.67% under the NP, 1/3M, and M treatments, respectively. In contrast, PAR decreased by 69.03%, 43.78%, and 50.52%, respectively, under the NP, 1/3M, and M treatments relative to CK. At the middle grain-filling stage, LAI under the different fertilization treatments increased by 93.83%, 44.83%, and 30.79%, respectively, compared with CK, whereas PAR decreased by 67.85%, 38.84%, and 41.85%, respectively. At the late grain-filling stage, compared with CK, LAI increased by 77.93%, 37.56%, and 14.55% under the NP, 1/3M, and M treatments, respectively, while PAR decreased by 66.36%, 54.94%, and 41.47%, respectively1.

**Figure 6:**
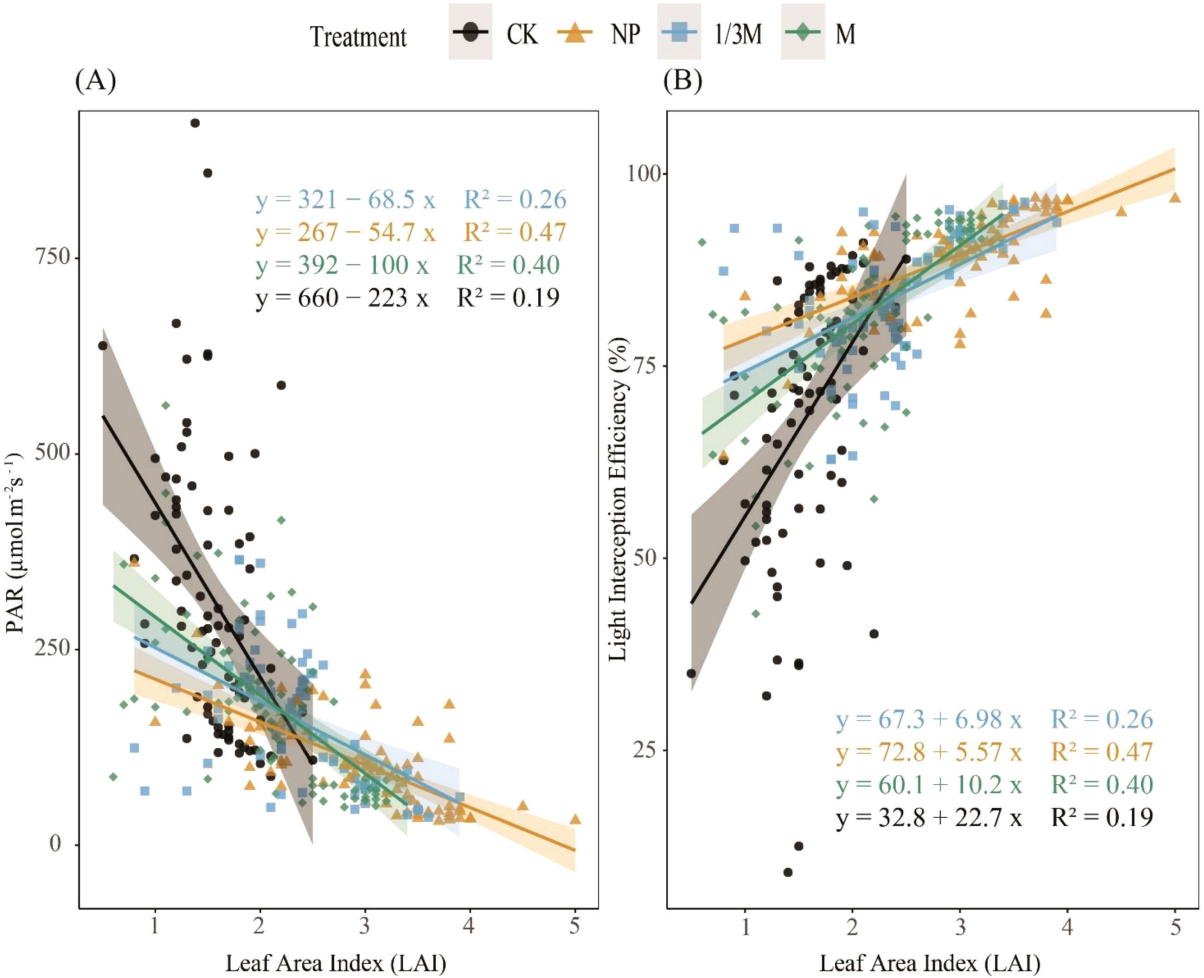
Relationships of leaf area index (LAI) with **(A)** photosynthetically active radiation (PAR) and **(B)** light interception efficiency under different fertilization treatments during the grain-filling period of maize: CK, no fertilization; NP, conventional chemical fertilization; 1/3M, optimized fertilization with one-third substitution by organic fertilizer; M, sole application of organic fertilizer. Solid lines indicate fitted linear regressions, and shaded areas represent the 95% confidence intervals.

As shown in **Figure 6**, maize LAI and canopy PAR under different fertilization treatments exhibited a consistently negative relationship: as LAI increased, transmitted PAR continuously decreased, indicating that canopy expansion significantly enhanced shading and reduced the amount of light reaching the lower canopy. The rate of decline differed among treatments, with the overall sensitivity ranked as CK > M > 1/3M > NP. In contrast, light interception efficiency increased with increasing LAI. Comparisons among treatments showed that the NP and 1/3M treatments maintained relatively high interception efficiency within the higher LAI range, reflecting a stronger canopy light interception capacity. The M treatment showed a smaller increase in interception efficiency, whereas CK, although showing an increasing trend with LAI, maintained a generally low interception level, suggesting that its canopy structure was relatively sparse and its capacity for light capture was limited.

### 3.3 Effects of different fertilization treatments on soil NCER, temperature sensitivity, and soil water content during the maize grain-filling period

**Figure 7:**
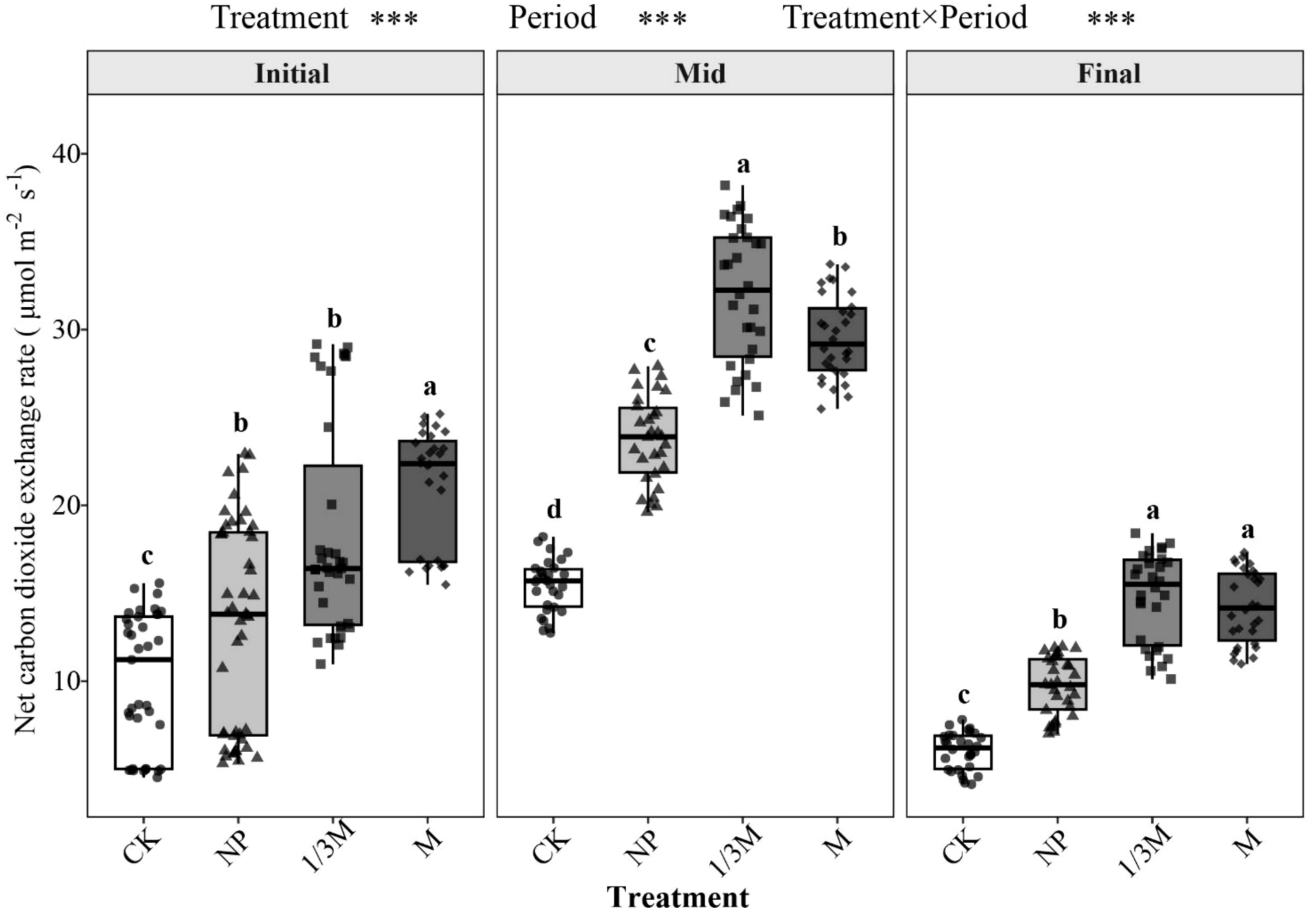
Effects of different treatments on the net soil CO_2_ exchange rate (NCER) during the maize grain-filling stage: CK, no fertilization; NP, conventional chemical fertilization; 1/3M, optimized fertilization with one-third substitution by organic fertilizer; M, sole application of organic fertilizer. Different lowercase letters indicate significant differences among treatments within the same period at *P* < 0.05. Asterisks above the panels indicate the significance of treatment, period, and treatment × period interaction; ***P < 0.001.

**Figure 7** shows that NCER under different treatments changed markedly during the maize grain-filling period, with values generally increasing as grain filling progressed, reaching a maximum at the middle grain-filling stage, and then declining at the late stage. At the early grain-filling stage, NCER was highest under the M treatment, followed by 1/3M and NP, whereas CK showed the lowest value. Compared with CK, NCER under NP, 1/3M, and M increased by 31.77%, 83.62%, and 110.2%, respectively. At the middle grain-filling stage, NCER further increased in all treatments, and the differences among treatments became more pronounced, with 1/3M showing a 107.3% increase relative to CK. At the late grain-filling stage, NCER declined across all treatments, but fertilization treatments still maintained higher levels than CK; compared with CK, NCER under 1/3M and M increased by 145.6% and 136.5%, respectively.

**Figure 8:**
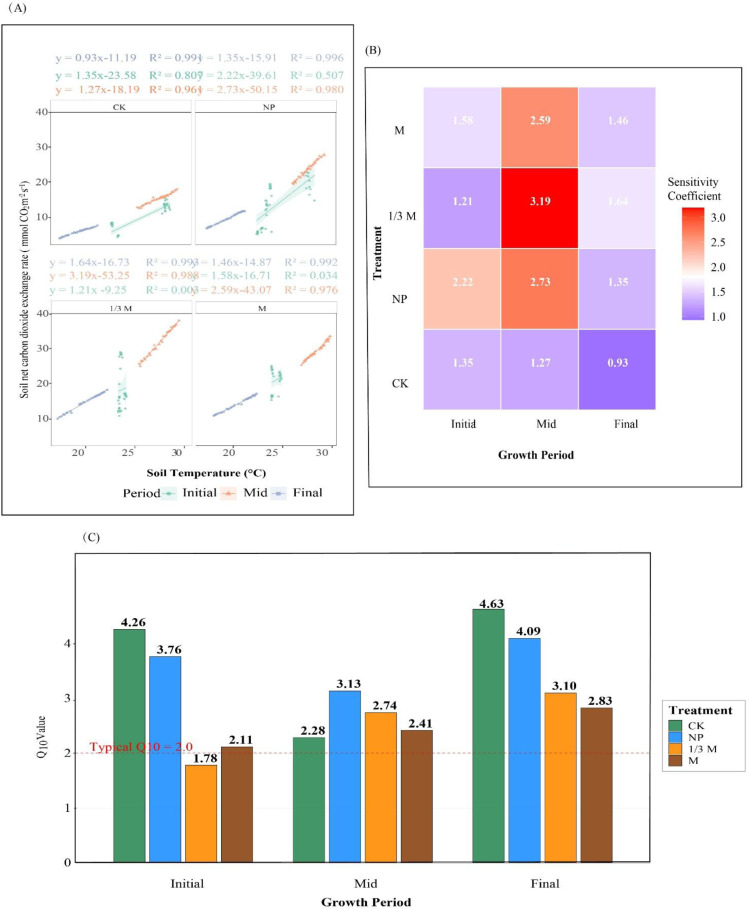
Temperature sensitivity of soil net carbon dioxide exchange rate (NCER) under different fertilization treatments during the grain-filling period of maize. **(A)** Linear relationships between NCER and soil temperature at the initial, middle, and final grain-filling stages under different fertilization treatments; **(B)** heatmap of sensitivity coefficients under different treatments and growth periods; and **(C)** Q_10_values under different fertilization treatments at different grain-filling stages. CK, no fertilization; NP, conventional chemical fertilization; 1/3M, optimized fertilization with one-third substitution by organic fertilizer; M, sole application of organic fertilizer. In **(A)**, solid lines indicate fitted regressions and shaded areas represent the 95% confidence intervals. In **(C)**, the dashed red line indicates the typical Q_10_value of 2.0.

**Figure 8** shows that soil NCER responded positively overall to increasing soil temperature, indicating that soil temperature was an important factor driving variation in soil CO_2_ release. Temperature sensitivity was jointly regulated by grain-filling stage and fertilization regime, with the middle grain-filling stage showing the greatest sensitivity. At this stage, the temperature sensitivity coefficients were 1.27 for CK, and 2.73, 2.59, and 3.19 for NP, M, and 1/3M, respectively, with 1/3M showing the highest sensitivity. At the late grain-filling stage, the temperature sensitivity coefficients generally declined across treatments, with values of 0.93 for CK, 1.35 for NP, 1.46 for M, and 1.64 for 1/3M, although all fertilization treatments still remained higher than CK. The Q_10_ results further indicated clear stage-dependent differences in the temperature response of soil respiration. At the early grain-filling stage, Q_10_ was relatively high under CK and NP, at 4.26 and 3.76, respectively, whereas M showed a value of 2.11 and 1/3M had the lowest value, 1.78. At the middle grain-filling stage, Q_10_ values were mainly concentrated within the range of 2-3. At the late grain-filling stage, Q_10_ values generally increased, with CK showing the highest value (4.63), followed by NP (4.09), whereas the corresponding values under 1/3M and M were 3.10 and 2.83, respectively.

**Figure 9:**
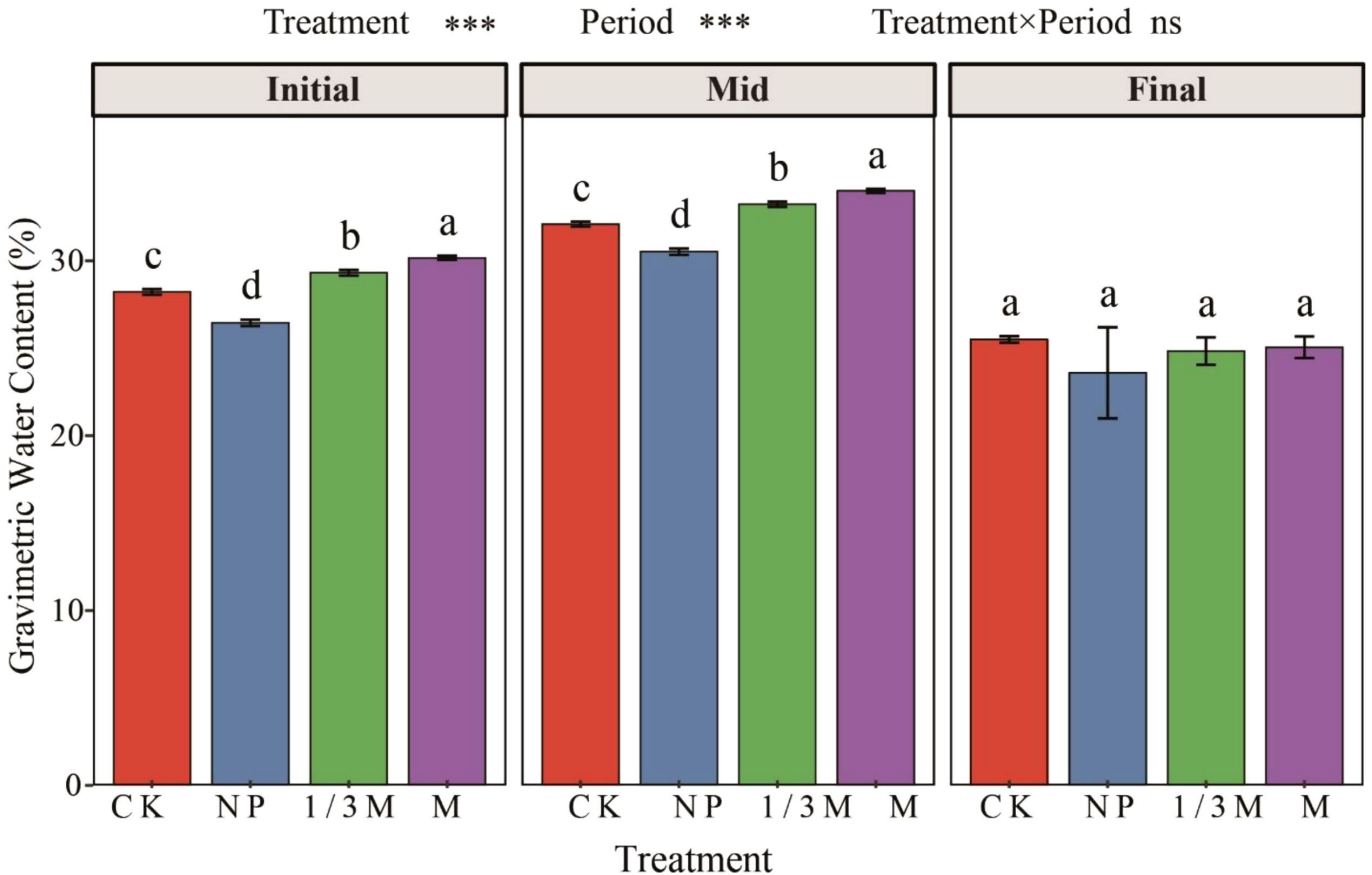
Changes in soil moisture content at different growth stages under different fertilization treatments: CK, no fertilization; NP, conventional chemical fertilization; 1/3M, optimized fertilization with one-third substitution by organic fertilizer; M, sole application of organic fertilizer. Different lowercase letters indicate significant differences among treatments within the same period at *P* < 0.05. Asterisks above the panels indicate the significance of treatment, period, and treatment × period interaction; ***P < 0.001.

Soil water content differed significantly among treatments at the early and middle grain-filling stages (**Figure 9**). Compared with CK, soil water content under the NP treatment was significantly lower at both stages, decreasing by 4.91%–6.25%, whereas both the 1/3M and M treatments significantly increased soil water content. The highest values were observed under the M treatment, with increases of 5.92%–6.90% relative to CK, followed by the 1/3M treatment, which increased by 3.53%–3.89%. By the late grain-filling stage, soil water content had declined overall across all treatments, and no significant differences were observed among treatments.

### 3.4 Effects of different fertilization treatments on leaf photosynthesis and transpiration characteristics during the maize grain-filling period

**Figure 10:**
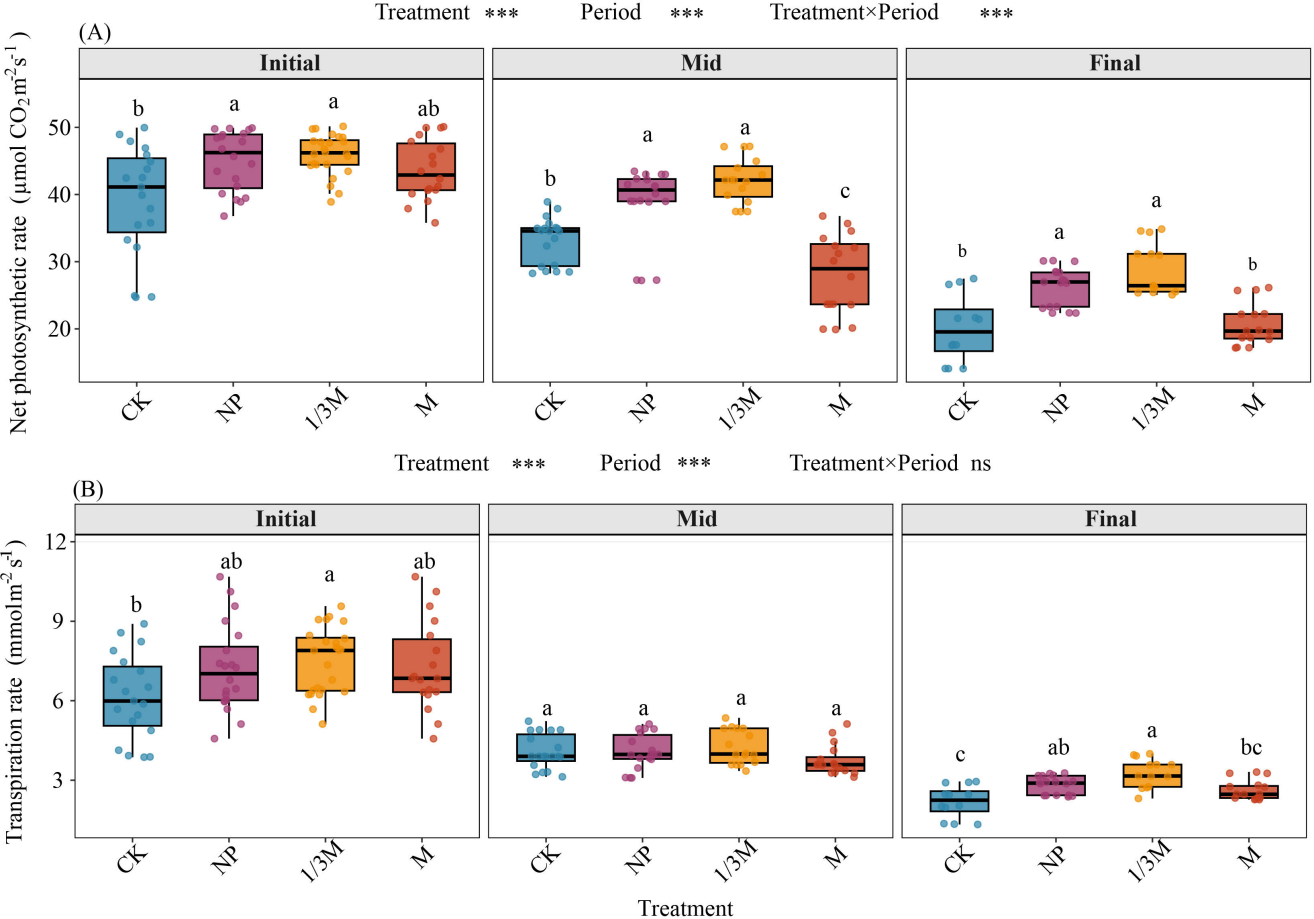
Changes and differences in net photosynthetic rate and transpiration rate of maize during the grain-filling stage under different: **(A)** Net photosynthetic rate at the initial, middle, and final grain-filling stages; **(B)** transpiration rate at the initial, middle, and final grain-filling stages. Initial, early grain-filling stage; Mid, middle grain-filling stage; Final, late grain-filling stage. CK, no fertilization; NP, conventional chemical fertilization; 1/3M, optimized fertilization with one-third substitution by organic fertilizer; M, sole application of organic fertilizer. Different lowercase letters indicate significant differences among treatments within the same period at *p* < 0.05. Asterisks above the panels indicate the significance of treatment, period, and treatment × period interaction; ns, not significant; ***p < 0.001.

Net photosynthetic rate (A) and transpiration rate (E) during the maize grain-filling period both declined significantly from the early to middle and late stages (**Figure 10**), indicating an overall weakening of leaf gas-exchange capacity as grain filling progressed. Taking the early grain-filling stage as the reference, the reductions in A at the late stage were 48.72% for CK, 41.55% for NP, 36.80% for 1/3M, and 52.59% for M. The corresponding reductions in E were 64.33%, 60.89%, 56.40%, and 64.06%, respectively, with 1/3M showing the smallest overall decline. At the early grain-filling stage, A under the NP and 1/3M treatments was significantly higher than that under CK, increasing by 15.11% and 17.38%, respectively, whereas A under the M treatment was intermediate and still 11.61% higher than that under CK. At the middle grain-filling stage, A under 1/3M and NP remained significantly higher than that under CK, by 26.80% and 16.71%, respectively, whereas A under M was significantly lower than that under CK, showing a decrease of 15.59% and indicating a marked mid-stage decline. At the late grain-filling stage, A declined overall across treatments, but 1/3M and NP still remained significantly higher than CK, by 44.67% and 31.21%, respectively, whereas M was comparable to CK, with only a 3.19% increase. Differences in E among treatments were stage-dependent. At the early grain-filling stage, E under 1/3M was significantly higher than that under CK, whereas NP and M were intermediate. No significant differences among treatments were observed at the middle grain-filling stage. At the late grain-filling stage, treatment differences became significant again, with 1/3M showing the highest E, followed by NP, while M and CK remained at relatively low levels. Overall, both 1/3M and conventional chemical fertilization (NP) were more effective in maintaining higher photosynthetic capacity during the middle and late grain-filling stages, whereas 1/3M showed a more pronounced advantage in sustaining transpiration at the early and late stages.

**Figure 11:**
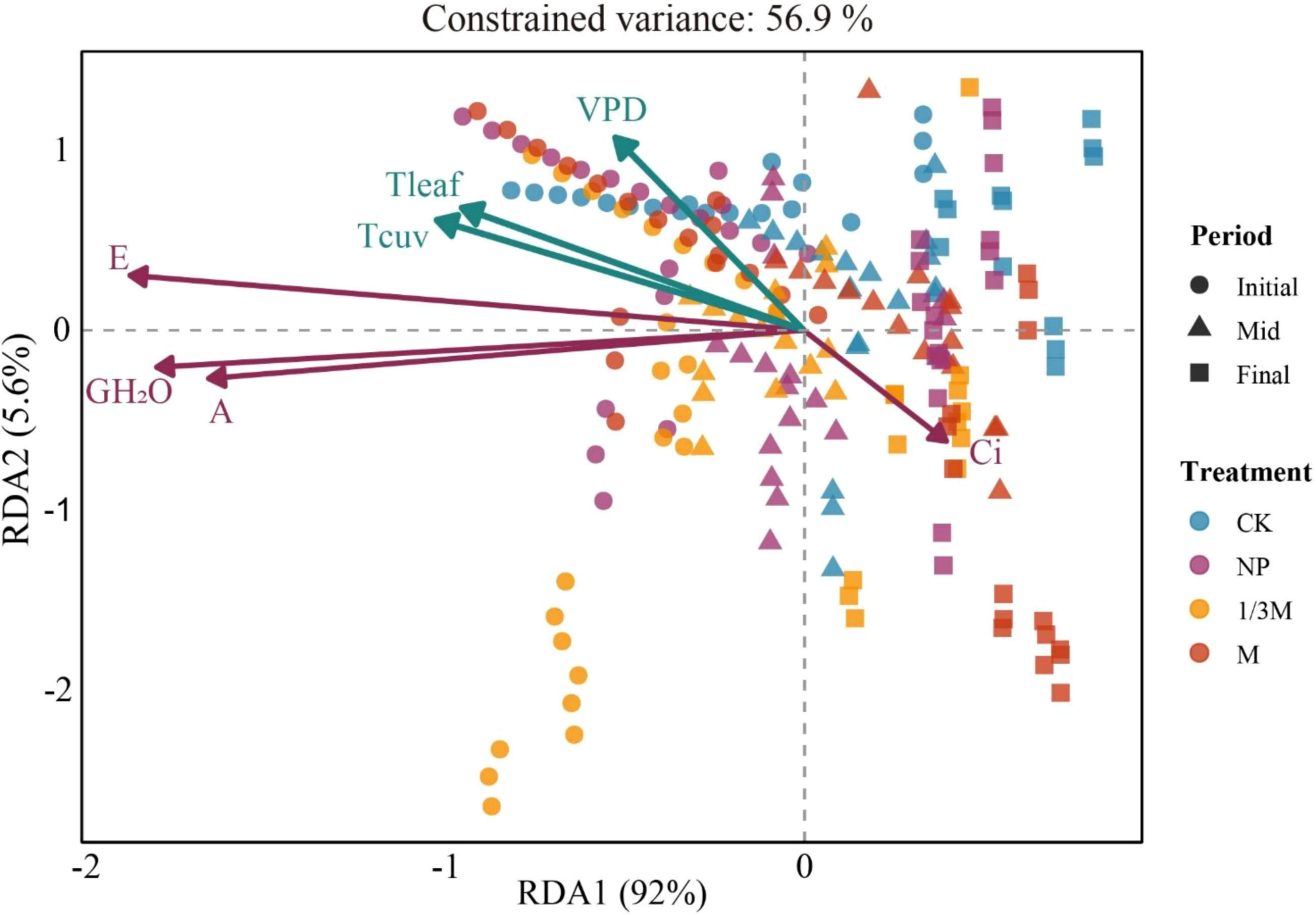
Redundancy analysis (RDA) of maize net photosynthetic rate with physiological and environmental factors (e.g., transpiration rate and stomatal conductance) during the grain-filling stage under different treatments: Initial, early grain-filling stage; Mid, middle grain-filling stage; Final, late grain-filling stage. CK, no fertilization; NP, conventional chemical fertilization; 1/3M, optimized fertilization with one-third substitution by organic fertilizer; M, sole application of organic fertilizer. A, net photosynthetic rate; E, transpiration rate; Ci, intercellular CO_2_concentration; GH_2_O, stomatal conductance to water vapor; Tleaf, leaf temperature; Tcuv, cuvette temperature; VPD, vapor pressure deficit.

Redundancy analysis (RDA) results (**Figure 11**) revealed the relationships of photosynthetic physiological traits with different fertilization treatments and grain-filling stages, with the model explaining 56.9% of the total constrained variance. RDA1 and RDA2 accounted for 92.0% and 5.6% of the constrained variance, respectively, and together explained 97.6% of the constrained variance, indicating that the ordination axes had very strong explanatory power for the relationships among variables. Differences among samples were mainly driven by the first ordination axis, RDA1.The sample distribution showed a clear separation among treatments and stages in the ordination space. Samples from the M treatment were mainly distributed on the right-hand side of the RDA1 axis, whereas those from the 1/3M treatment were primarily clustered on the left-hand side. Samples from the CK and NP treatments were mostly located in the upper central part of the ordination plot, suggesting that fertilization treatment was one of the key factors driving sample differentiation. Overall, some samples from the 1/3M treatment tended to be distributed in the same direction as A, E, and GH_2_O, whereas CK samples were more closely associated with the direction of Ci, indicating that photosynthetic performance under CK was relatively constrained. In particular, samples from the M treatment showed a more distinct clustering or shift at the late grain-filling stage, suggesting that its physiological status differed from that of the other treatments during the later stage of grain filling. At the same time, samples from different grain-filling stages (Initial, Mid, and Final) also showed a certain degree of separation, indicating that physiological senescence and environmental changes during grain filling further influenced the expression of differences among fertilization treatments.

### 3.5 Effects of different fertilization treatments on nutrient uptake and allocation in maize

**Figure 12:**
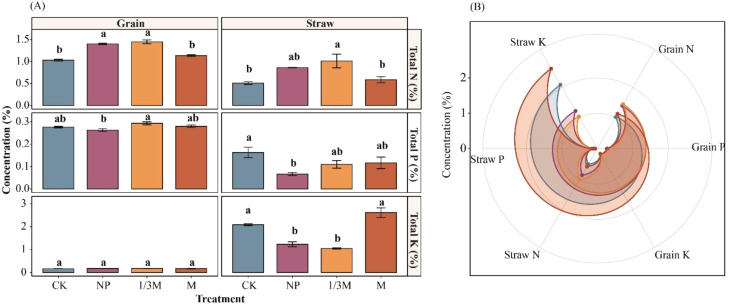
Effects of different fertilization treatments on N, P, and K contents in maize grains and stems: **(A)** Concentrations of total nitrogen (N), total phosphorus (P), and total potassium (K) in grain and straw under different fertilization treatments; **(B)** radar chart showing the distribution patterns of N, P, and K concentrations in grain and straw among treatments. CK, no fertilization; NP, conventional chemical fertilization; 1/3M, optimized fertilization with one-third substitution by organic fertilizer; M, sole application of organic fertilizer. Bars represent the mean ± standard error. Different lowercase letters indicate significant differences among treatments within the same organ at *p* < 0.05.

As shown in **Figure 12**, different fertilization treatments significantly altered the N, P, and K contents and their allocation between maize grain and straw. Among the grain nutrients, N showed the most stable response to fertilization treatments, with grain N under the 1/3M and NP treatments increasing by 40.30% and 35.80%, respectively, compared with CK. Differences in grain P were relatively small, although the highest value was observed under 1/3M and the lowest under NP. Grain K varied only slightly among treatments, ranging from 0.17% to 0.18%.For straw nutrients, straw N under the 1/3M treatment increased by 98.70% relative to CK, followed by increases of 69.30% under NP and 15.00% under M. In contrast, straw P was highest under CK and generally declined after fertilization, with decreases of 59.20%, 32.70%, and 28.60% under NP, 1/3M, and M, respectively, compared with CK. Straw K under the M treatment increased significantly by 25.20% relative to CK, whereas it decreased by 40.90% and 49.80% under NP and 1/3M, respectively.

### 3.6 Effects of different fertilization treatments on maize yield and its components

**Figure 13:**
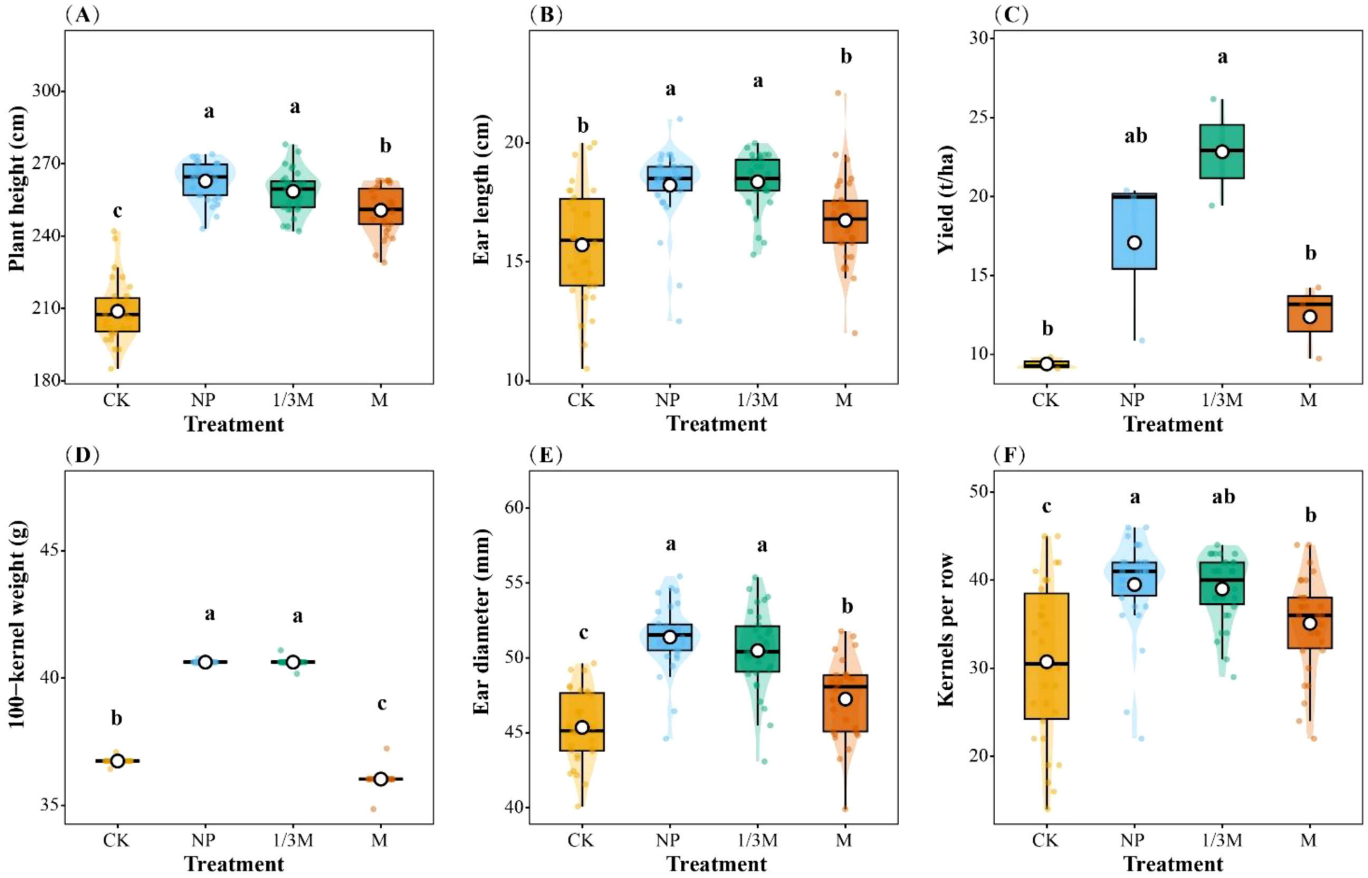
Effects of different fertilization treatments on maize plant morphology and yield-related traits: **(A)** Plant height; **(B)** ear length; **(C)** grain yield; **(D)** 100-kernel weight; **(E)** ear diameter; and **(F)** kernels per row. CK, no fertilization; NP, conventional chemical fertilization; 1/3M, optimized fertilization with one-third substitution by organic fertilizer; M, sole application of organic fertilizer. Different lowercase letters indicate significant differences among treatments at *p* < 0.05.

Different fertilization treatments significantly improved maize agronomic traits and yield components (**Figure 13**). Overall, maize under the NP and 1/3M treatments showed better agronomic performance than that under the M and CK treatments. Plant height differed significantly among treatments, with increases of 25.86%, 23.80%, and 20.05% under NP, 1/3M, and M, respectively, compared with CK. Ear length was also most markedly increased under the NP and 1/3M treatments, rising by 15.98% and 16.87%, respectively, relative to CK. Ear diameter and grain number showed greater sensitivity to fertilization treatments. Compared with CK, ear diameter increased significantly by 13.30% under NP, 11.31% under 1/3M, and 4.19% under M. The number of grains per row increased by 28.54% and 26.91% under NP and 1/3M, respectively, while M also showed a significant increase of 14.22%. The 100-grain weight showed clear variation among treatments: both NP and 1/3M increased by 10.56% relative to CK, whereas M was slightly lower than CK, decreasing by 1.93%. Final grain yield was highest under the 1/3M treatment, with an increase of 143.0% relative to CK; yield under NP increased by 81.76%, while that under M increased by 31.76%.

**Figure 14:**
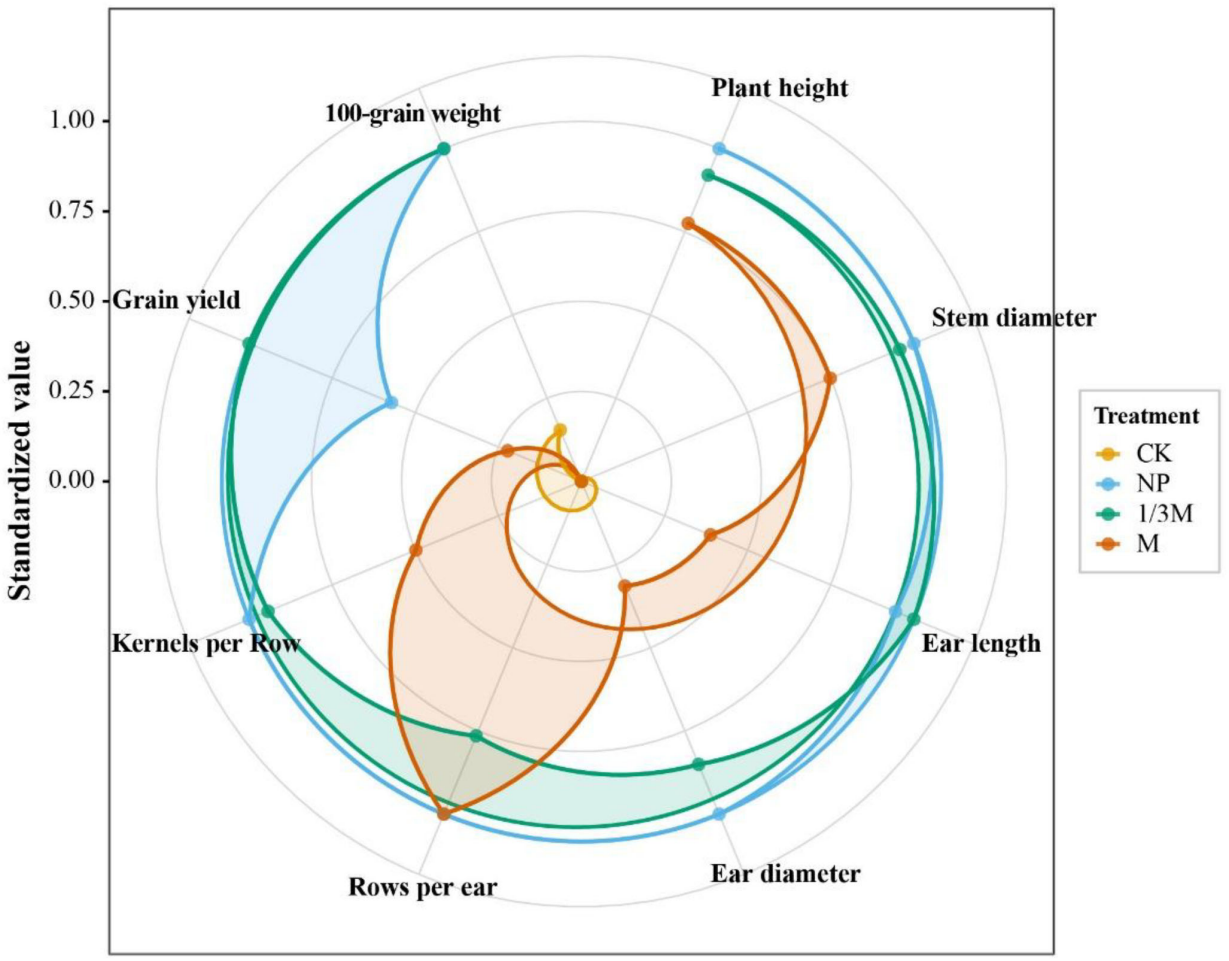
Standardized radar chart of maize growth, ear traits, and yield under different fertilization treatments: Traits included plant height, stem diameter, ear length, ear diameter, rows per ear, kernels per row, grain yield, and 100-grain weight. CK, no fertilization; NP, conventional chemical fertilization; 1/3M, optimized fertilization with one-third substitution by organic fertilizer; M, sole application of organic fertilizer.

As shown in **Figure 14**, the standardized values of individual traits were used for a comprehensive comparison of yield-related agronomic traits and yield indicators under different fertilization treatments. Overall, the curves for the NP and 1/3M treatments extended further outward and covered a larger area, indicating a higher overall performance across most traits. By contrast, the CK curve was located closest to the center, reflecting relatively low values for plant height, stem diameter, ear morphology, and yield-related traits. The overall performance of the M treatment was intermediate between those of the NP and 1/3M treatments. For individual traits, the NP treatment showed more pronounced advantages in plant height, stem diameter, and structural traits such as ear length and ear diameter. The 1/3M treatment performed better in key yield-formation traits, including 100-grain weight and grain yield, and was also comparable to the NP treatment in several ear and grain traits. In contrast, the M treatment showed a more pronounced outward extension for row number, but its overall performance in ear-grain composition and yield-related traits was weaker than that of NP and 1/3M. Both NP and 1/3M were able to improve agronomic traits and yield components more evenly, thereby achieving superior overall performance.

### 3.7 Synergistic relationships among soil properties, physiological traits, and yield-related characteristics

**Figure 15:**
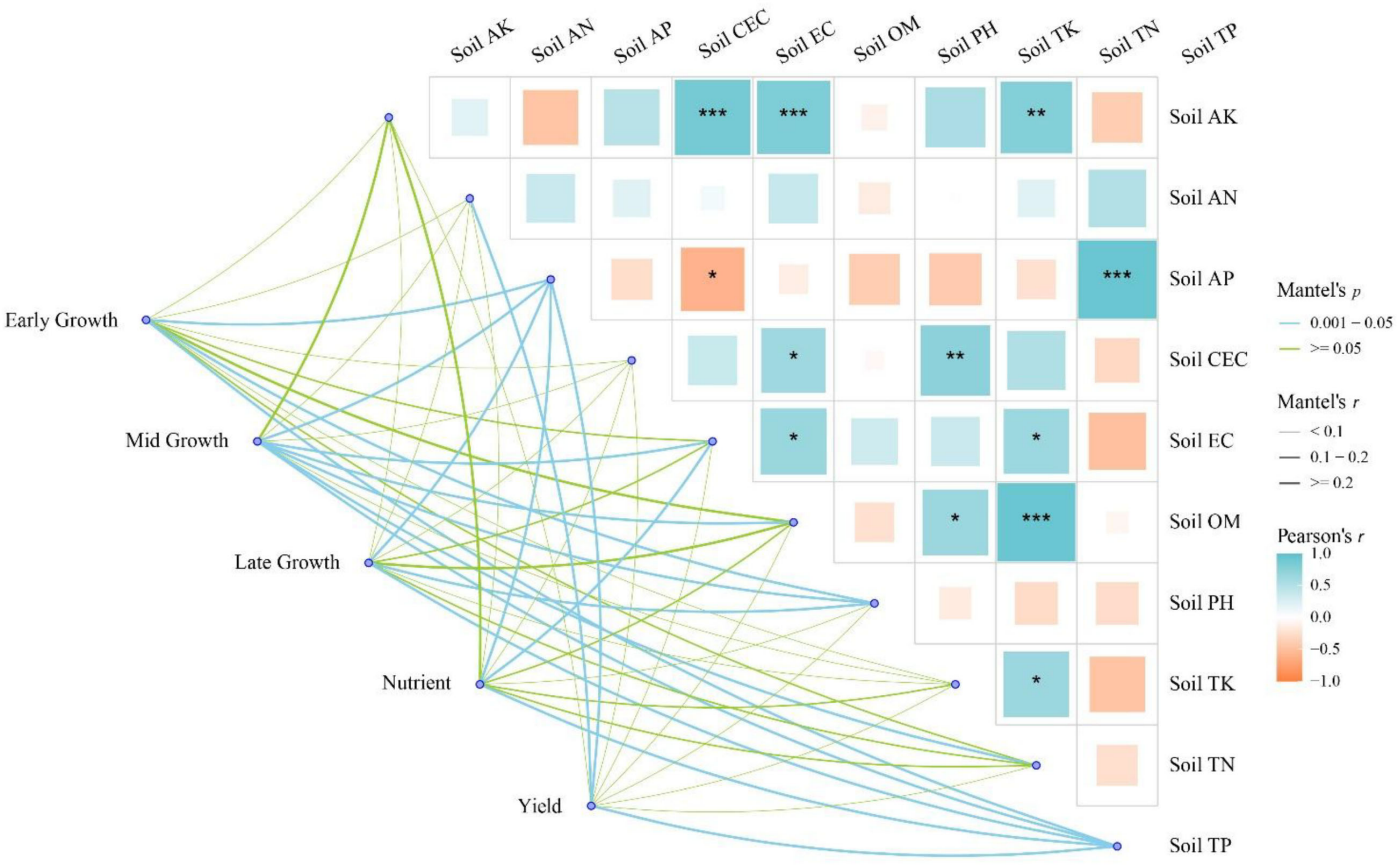
Mantel correlation heatmap of soil properties, canopy physiology, soil respiration, and yield traits. The right panel shows Pearson correlation coefficients for 10 soil environmental factors, with the color gradient indicating correlation strength (blue = positive correlation, orange = negative correlation). Connecting lines on the left, derived from Mantel tests, illustrate the associations between five trait modules and the soil factor matrix. Trait modules include: Early Growth (early-stage functional traits), Mid Growth (mid-stage functional traits), Late Growth (late-stage functional traits), Plant Nutrient Traits, and Yield & Yield Component Traits. Line thickness scales with Mantel’s r statistic; line color indicates significance level (*p* < 0.001, 0.001 ≤ *p* < 0.05, *p* ≥ 0.05 [ns, not significant]). Significant correlations in the heatmap are marked with asterisks (*p < 0.05, **p < 0.01, ***p < 0.001).

Mantel module network analysis was performed to examine the relationships between 10 soil factors and five trait modules. Overall, the results indicated that all five modules were significantly linked with some soil factors (**Figure 15**; *p* < 0.05), and all significant links had Mantel’s r values ≥ 0.2. The early grain-filling module showed significant associations (*p* < 0.05) with three soil factors, namely Soil AP, Soil pH, and Soil TP, and all three links had r ≥ 0.2. In addition, one non-significant link with r ≥ 0.2 was observed for Soil OM (r = 0.222, *p* = 0.118). The middle grain-filling module exhibited significant associations (*p* < 0.05) with six soil factors, including Soil AP, Soil EC, Soil OM, Soil pH, Soil TN, and Soil TP, and all six significant links had r ≥ 0.2. One additional non-significant link with r ≥ 0.2 was found for Soil AK (r = 0.235, *p* = 0.057). Therefore, the middle grain-filling module showed a total of seven links with r ≥ 0.2, of which six were significant. The late grain-filling module showed significant associations (*p* < 0.05) with three soil factors, namely Soil AP, Soil pH, and Soil TP, and all of these links had r ≥ 0.2. One additional non-significant link with r ≥ 0.2 was observed for Soil OM (r = 0.212, *p* = 0.079). The physiological trait module was significantly associated (*p* < 0.05) with three soil factors, including Soil AP, Soil EC, and Soil TP, and all of these links had r ≥ 0.2. One further non-significant link with r ≥ 0.2 was found for Soil AK (r = 0.242, *p* = 0.053). The yield trait module showed significant associations (*p* < 0.05) with three soil factors, namely Soil AN, Soil AP, and Soil TP, and all of these links also had r ≥ 0.2. No additional non-significant links with r ≥ 0.2 were observed for this module.

## 4 Discussion

### 4.1 Effects of different fertilization regimes on soil nutrients and water status

The present study showed that soil pH remained stable within the range of 8.54–8.67 across treatments, indicating that long-term differences in fertilization regime had a relatively limited effect on soil pH in the continuous maize cropping system on carbonate-rich Sierozems. This finding is consistent with previous studies on CO_2_ release and carbonate system dynamics in calcareous soils (Cai et al., 2018; Sun et al., 2023). The limited variation in soil pH may also be related to the strong buffering capacity of Sierozems, in which soil pH is strongly regulated by CaCO_3_ equilibria and is therefore generally less responsive to long-term fertilization management (Bontpart et al., 2024). Different fertilization regimes primarily led to divergence in the structure of the soil nutrient pool. The NP treatment significantly enhanced the soil phosphorus pool (Xue et al., 2013), with available phosphorus (AP) and total phosphorus (TP) increasing by 128.8% and 27.52%, respectively, relative to CK.

In contrast, the M treatment mainly increased soil organic matter (SOM; +27.36% vs. CK), total nitrogen (TN; +24.60%), and available potassium (AK; +99.03%), while also increasing electrical conductivity (EC; +10.04%). These changes may be related to the nutrient composition and release characteristics of the organic fertilizer itself; meanwhile, the input of soluble ions from organic fertilizer may also have contributed to the increase in EC, especially in calcareous soils under irrigation where solute redistribution is more active (Moradi et al., 2024). On the other hand, long-term chemical fertilizer input enlarged the soil phosphorus pool (Yan et al., 2016) but at the same time reduced soil water content during the early and middle grain-filling stages (Liu et al., 2013, Liu et al., 2023a; 2023b), indicating that conventional chemical fertilization neither improved soil structure nor reduced water consumption. By contrast, long-term organic matter input under organic fertilizer treatment enhanced soil water-holding capacity (**Figure 9**). This improvement is likely attributable to the role of organic inputs in increasing SOM, improving aggregation, and enhancing soil moisture retention, which has also been reported in other irrigated cropping systems receiving organic fertilizer. However, the beneficial effects of M on soil fertility and water retention do not necessarily translate directly into better crop performance, because sole organic fertilizer application may simultaneously increase the concentration of soluble salts in the root zone and create a less favorable osmotic environment for root water uptake, particularly under irrigation conditions. In this case, improved soil quality at the bulk-soil level may coexist with greater ionic or salt-related stress around the roots. Similar studies under irrigated conditions have shown that organic fertilizer can improve soil fertility and moisture-related properties, while also being associated with increased EC or greater sensitivity to the salt environment depending on fertilizer composition and field conditions (Khan et al., 2022b; Salman et al., 2023). However, the higher EC values suggest that organic fertilizer may introduce greater amounts of soluble ions, while irrigation-driven solute migration and accumulation may increase the risk of osmotic stress to crops (Ding et al., 2020, Liu et al., 2023b). Therefore, although the M treatment showed advantages in improving soil fertility and water retention, it may also have increased ion concentrations in the crop root-zone environment, thereby imposing stress on plant growth (Mohanavelu et al., 2021). It should also be noted that not all soil properties showed significant changes after eight years of fertilization. In particular, the non-significant responses of cation exchange capacity (CEC) and total potassium (TK) may be because these properties are more strongly influenced by the combined effects of mineral composition, soil texture, inherent soil organic matter status, and crop uptake, and are therefore generally less sensitive than labile nutrient pools to fertilization management (Ma et al., 2024; Bontpart et al., 2024).

In the PCA ordination space, the 1/3M treatment was positioned between NP and M, and simultaneously increased AP (+80.85%), TP (+16.25%), and AK (+22.27%) relative to CK. This indicates that partial substitution of chemical fertilizer with organic fertilizer in the Sierozem system of the Yellow River irrigated area can simultaneously support rapid nutrient supply and long-term improvement in soil fertility. This is likely because chemical fertilizer ensures the timely supply of readily available nutrients, whereas sole organic fertilizer application tends to release nutrients more slowly; at the same time, organic fertilizer can alleviate the soil structure deterioration and increased water-consumption risk associated with sole chemical fertilization (Zhang et al., 2018; Chen et al., 2024). Moreover, the overall nutrient performance of the 1/3M treatment was not reflected in an outstanding improvement in any single trait, but rather in a more balanced and coordinated enhancement across multiple traits. Compared with the NP treatment, 1/3M introduced additional organic matter and therefore had greater potential to improve soil fertility and water retention; compared with the M treatment, it reduced the potential risk of root-zone stress associated with increased EC (Ding et al., 2020, Liu et al., 2023b). Therefore, under the conditions of border irrigation on Sierozems in the Yellow River irrigated area, partial substitution with organic fertilizer is more likely to represent an optimized soil fertility management strategy that helps reduce the vulnerability of the farmland system (Wang et al., 2025a).

### 4.2 Effects of different fertilization treatments on maize canopy function and photosynthetic characteristics

Across all fertilization treatments, both net photosynthetic rate (A) and transpiration rate (E) declined as grain filling progressed. However, the magnitude of decline was smallest under the 1/3M treatment, with A and E decreasing by 36.80% and 56.40%, respectively, relative to the early grain-filling stage. The NP treatment showed the second smallest decline, with reductions of 41.55% in A and 60.89% in E, whereas the M treatment exhibited the greatest decline in A, decreasing by 52.59% relative to the early grain-filling stage (**Figure 1**). Comparisons among treatments during grain filling further showed that, at the middle grain-filling stage, A under the 1/3M and NP treatments was higher than that under CK, by 26.80% and 16.71%, respectively, whereas A under the M treatment was lower than that under CK (−15.59%). This suggests that sole application of organic fertilizer may be insufficient to meet nutrient demand during the middle grain-filling stage of maize (Fan et al., 2021; Imran, 2024; Wang et al., 2025b). Changes in photosynthesis during grain filling were mainly regulated by stomatal processes (Li et al., 2026), which is consistent with previous findings showing coordinated variation among maize photosynthetic rate, stomatal conductance, and transpiration (Khamis, 2020; Guo et al., 2022). In addition, A was negatively correlated with vapor pressure deficit (VPD) and ambient CO_2_ concentration (Ca) during the middle and late grain-filling stages, while its relationship with temperature shifted from positive at the early stage to negative at the middle stage. This indicates that under the high-temperature and high-evaporation conditions of the middle grain-filling stage, the interaction between stomatal limitation and heat stress may determine differences in canopy function and physiological performance among treatments (Guo et al., 2022). Soil water content data showed that the NP treatment reduced soil water content during the early and middle grain-filling stages, by 4.91%-6.25% relative to CK, whereas both the 1/3M and M treatments increased soil water content, by 3.53%-3.89% and 5.92%-6.90%, respectively. In irrigated farmland, alternating wetting and drying cycles associated with irrigation affect soil organic matter mineralisation and humification processes, as well as crop physiological performance. Soil moisture status influences not only plant stomatal behavior, but also the rhizosphere environment and gas diffusion (Gao et al., 2021; Avila et al., 2023). Therefore, the better water-holding capacity under the 1/3M treatment may have alleviated stomatal closure and thereby helped sustain grain filling during the later stages. Although soil water content was also high under the M treatment, the decline in A at the middle grain-filling stage indicates a limitation of sole organic fertilizer application in terms of nutrient supply. Across all three grain-filling stages, SPAD values remained highest under the NP and 1/3M treatments, increasing by 118.77%-137.5% relative to CK. This suggests that a more stable leaf N status can delay the decline in crop physiological function. By contrast, the increase in SPAD under the M treatment was much smaller (+21.66%-36.80%), which may be attributed to the slower nutrient release from sole organic fertilizer application and its mismatch with crop nutrient demand (Fan et al., 2021; Imran, 2024).

### 4.3 Effects of different fertilization treatments on soil NCER during the maize grain-filling period

Soil NCER under different fertilization regimes reached its maximum at the middle grain-filling stage and declined at the late stage (**Figure 4**) (Hirte et al., 2018). Fertilization treatments increased soil NCER. Relative to CK, the 1/3M treatment increased NCER by 83.62%, 107.3%, and 145.6% at the early, middle, and late grain-filling stages, respectively, whereas the M treatment increased NCER by 110.19% at the early stage and by 136.5% at the late stage. The correlations between NCER and both A and E became stronger during the middle and late grain-filling stages, indicating a coordinated relationship between soil CO_2_ exchange and crop carbon assimilation and transpiration processes. This may be attributed to enhanced allocation of assimilated carbon belowground, which in turn stimulated soil CO_2_ exchange (Liang et al., 2021). The 1/3M treatment still maintained a relatively high increase in soil NCER at the late grain-filling stage, which was consistent with its higher A than CK at the same stage (+44.67%). This further supports the existence of mutual reinforcement and coordination between soil CO_2_ exchange and plant photosynthetic and respiratory processes. Overall, soil NCER responded positively to increasing soil temperature, and its temperature sensitivity was greatest at the middle grain-filling stage. This may be because increasing temperature enhanced substrate supply and biological activity, thereby making the soil respiration process more sensitive to temperature. The Q_10_ values under different fertilization treatments ranged from 2 to 3 during grain filling, indicating that temperature sensitivity was not determined by temperature alone (Chen et al., 2020), but was jointly regulated by soil water content, substrate availability, and crop physiological status (Sun et al., 2023).

### 4.4 Effects of different fertilization treatments on soil NCER during the maize grain-filling period

The present study demonstrated significant differences in yield among fertilization treatments. Compared with the control, yield increased by 143.0% under the 1/3M treatment, by 31.76% under the M treatment, and by 81.76% under the NP treatment. Moreover, compared with NP, the 1/3M treatment further increased yield by 33.69% despite the already high yield level achieved under NP. These results indicate that mineral nutrient inputs can substantially improve maize yield, whereas partial substitution of chemical fertilizer with organic fertilizer does not simply increase the total nutrient supply, but more likely achieves additional yield gains by improving key physiological processes during grain filling and enhancing grain-filling efficiency (Zhang et al., 2018; Fan et al., 2021; Wang et al., 2025b).

From the perspective of yield components, both the NP and 1/3M treatments improved maize ear traits and increased 100-grain weight, indicating that both fertilization regimes promoted both grain number formation and grain weight formation. However, a clear difference in final yield was still observed between the two treatments. This is likely because organic fertilizer substitution enhanced the persistence of grain filling during the middle and late stages. Compared with NP, the 1/3M treatment maintained a higher net photosynthetic rate and transpiration level at the late grain-filling stage, while SPAD and LAI were also more stable. This suggests that partial organic fertilizer substitution provided a stronger assimilate supply capacity during the rapid grain-filling and late grain-filling stages (Li et al., 2018; Duan et al., 2024). Previous studies have shown that yield improvement in maize depends more strongly on the maintenance of functional leaves and the continuous supply of assimilates during the middle and late grain-filling stages than on temporary advantages in vegetative growth or early canopy expansion (Guo et al., 2021; Duan et al., 2024). Therefore, the yield advantage of the 1/3M treatment is more likely to be attributed to a longer duration of source activity during the later grain-filling period (Deng et al., 2023), rather than simply to a larger canopy structure.

In addition, the better water-holding capacity observed under the 1/3M treatment indicates that partial organic input improved root-zone water status. In irrigated maize systems, soil water status often determines nitrogen fertilizer efficiency and influences assimilate supply during grain filling and final grain weight through stomatal regulation in crop leaves (Sandhu et al., 2019; Li et al., 2024). In the present study, net photosynthetic rate was highly positively correlated with stomatal conductance (GH_2_O) throughout all three grain-filling stages, indicating that stomatal limitation is an important pathway restricting assimilate production during grain filling. Therefore, the more favorable soil moisture conditions under the 1/3M treatment during grain filling may have reduced the probability of stomatal closure, thereby delaying photosynthetic decline, allowing more assimilates to be allocated to the grain, and ultimately increasing yield (Efthimiadou et al., 2010; Khamis, 2020).

Although the M treatment increased yield by 31.76%, and improved soil water content (**Figure 7**) as well as soil OM, TN, and AK (**Figure 15**), photosynthetic rate declined markedly during the middle grain-filling stage (**Figure 1**), and 100-grain weight decreased rather than increased (**Figure 11**). This suggests that sole application of organic fertilizer may have limitations related to asynchronous nitrogen release relative to crop nutrient demand during grain filling, as well as constraints imposed by elevated EC on physiological maintenance during this period. As a result, although soil properties were improved, these improvements were not effectively translated into yield advantages (Liang et al., 2021; Imran, 2024). Therefore, under border-irrigated Sierozem conditions, the key to yield improvement lies not only in increasing the size of the soil nutrient pool, but also in maintaining canopy function during the middle and late grain-filling stages and enhancing grain filling through the coordination of soil water-holding capacity and nutrient supply timing (Zhang et al., 2018; Sandhu et al., 2019).

### 4.5 Mantel correlation analysis of trait modules under different fertilization regimes

Different fertilization regimes altered soil nutrient supply and the physicochemical environment, thereby affecting physiological processes and nutrient translocation at different stages of grain filling, which ultimately resulted in differences in yield. To explore, at an integrated level, the coordinated relationship among soil nutrients, grain-filling physiological processes, and yield formation, this study combined Pearson correlation heatmap analysis with a Mantel module network analysis (**Figure 15**). The Pearson heatmap was used to characterize the coordinated variation structure among soil factors, whereas the Mantel network was used to evaluate the overall associations between soil factors and five trait modules. Soil fertility was not driven by a single indicator, but rather by the combined effects of multiple soil ions that jointly regulated maize grain filling, nutrient allocation, and final yield formation. The Pearson correlation heatmap showed significant correlations of soil organic matter (OM) with soil N, P, and K, indicating that long-term fertilization exerts a systematic reshaping effect on the soil environment (Gai et al., 2018; Lu, 2020; Wang et al., 2020). However, the relatively weak correlations between soil organic matter (SOM) and yield- or nutrient-related variables in the present study may be related to the specific properties of this soil system. SOM is a relatively stable and integrative background soil property, whereas grain yield and plant nutrient accumulation are more directly regulated by in-season nutrient availability, soil water status, and maize physiological performance during growth. Previous studies have shown that the relationship between SOM and crop yield is highly context dependent, and at the field scale it is often weak or inconsistent because it is jointly influenced by soil type, climate, and water and fertilizer management practices (Oldfield et al., 2019). In calcareous soils, this inconsistency may be even more pronounced because high CaCO_3_ content and alkaline pH can alter nutrient phytoavailability and plant uptake processes; therefore, an increase in bulk SOM does not necessarily translate directly into proportional increases in crop nutrient accumulation or yield (Bontpart et al., 2024). In the Mantel network, the physiological trait modules at the early, middle, and late grain-filling stages were all centrally and associated with OM, AN, AP, AK, TN, TP, and TK. In addition, the plant nutrient accumulation and organ allocation modules, as well as the yield trait module, were also linked to multiple soil factors. These results reveal a coordinated network of soil–physiological process–allocation–yield, suggesting that yield differences among fertilization treatments were mainly driven by fertilizer-induced changes in soil nutrient content and the effective supply capacity of the soil nutrient pool, which then influenced canopy functional structure and assimilate supply during the critical grain-filling period, thereby altering grain filling and final yield (Gai et al., 2018; Lu, 2020; Wang et al., 2020).

Mechanistically, increased SOM enhances soil aggregate stability and water-holding capacity, and promotes nutrient transformation through the combined effects of mineralisation, humification, and microbial activity. This improves the in-season availability of AN, AP, and AK, thereby facilitating the maintenance of higher SPAD, LAI, and photosynthetic activity during the middle and late grain-filling stages, ensuring a sustained assimilate supply from functional leaves and supporting continued grain filling, grain weight formation, and yield improvement (Tisdall and OADES 1982, Mustafa et al., 2020; Wang et al., 2020). By contrast, sole reliance on chemical fertilizer tends to favor rapid nutrient supply but is less capable of sustaining nutrient availability over time, thereby accelerating functional leaf senescence and ultimately restricting grain filling and yield formation (Tisdall and Oades, 1982). In addition, Sierozems generally have relatively high pH, which confers a strong P-fixation capacity. Therefore, an increase in TP does not necessarily translate into a proportional increase in plant-available P; rather, continuous crop uptake depends more strongly on the content of available P and the desorption and supply capacity of soil P. The degree of P availability may therefore influence grain filling in maize to some extent (Tunesi et al., 1999; Brownrigg et al., 2022).

At the same time, the significant coordination of K with the grain-filling modules, yield module, and plant nutrient module suggests that increased K promotes grain filling through a chain of effects involving transport efficiency, grain-filling persistence, and 100-grain weight. This is attributable to the crucial role of K in assimilate transport. During maize grain filling, K not only reflects whether soil nutrient supply is adequate, but also indicates its important role in regulating assimilate transport and source–sink coordination. Previous studies have shown that K^+^ is one of the major cations in the phloem and can promote sugar loading and long-distance transport, thereby supporting the efficiency of assimilate translocation from leaves to grains and other organs (Dreyer et al., 2017). K therefore affects not only water relations and photosynthesis at the source, but also the transport efficiency of assimilates required for grain filling at the sink (Lemoine et al., 2013).

Overall, partial substitution of chemical fertilizer with organic fertilizer is more likely to achieve a better balance between improving soil fertility and maintaining stable yield, whereas complete substitution may, under some conditions, be constrained by asynchronous nutrient release, environmental risks, or unstable yield performance. Therefore, in combination with the network analysis shown in **Figure 15**, the treatment most conducive to yield improvement is not the one that simply maximizes the content of a single nutrient element, but rather the one that simultaneously increases SOM and provides a more balanced in-season supply of AN, AP, and AK during grain filling, while using the pool capacities of TP, TN, and TK as coordinated reserve support to sustain both source and sink functions during the middle and late grain-filling stages.

## 5 Conclusion

In this study, the 1/3M treatment showed a stronger coordinated regulatory effect on plant-soil carbon exchange processes. Different fertilization regimes also affected nutrient allocation between grain and straw, with clear organ-specific patterns. Overall, in the irrigated continuous maize cropping system on Sierozem in the Yellow River irrigated area of Gansu Province, one-third substitution of chemical fertilizer with organic fertilizer was more effective in simultaneously improving soil fertility, sustaining canopy function during grain filling, stabilizing soil carbon processes, and increasing yield. This treatment therefore represents a more promising long-term fertilization strategy for the region. Further research integrating different organic fertilizer sources, substitution ratios, and full-growth-period observations in arid regions, together with economic benefit analysis, would help to refine the mechanistic understanding of how fertilization regulates soil-plant carbon and nitrogen coordination and yield formation, while also providing a stronger basis for evaluating the scientific soundness, economic feasibility, and wider applicability of this fertilization strategy as a long-term regional management option.

## Data Availability

The original contributions presented in the study are included in the article. Further inquiries can be directed to the corresponding author.
